# A taxonomic assessment of novel and remarkable fungal species in *Didymosphaeriaceae* (*Pleosporales, Dothideomycetes*) from plant litter

**DOI:** 10.3389/fmicb.2022.1016285

**Published:** 2022-11-22

**Authors:** Danushka S. Tennakoon, Kasun M. Thambugala, Nimali I. de Silva, Nakarin Suwannarach, Saisamorn Lumyong

**Affiliations:** ^1^Research Center of Microbial Diversity and Sustainable Utilization, Faculty of Science, Chiang Mai University, Chiang Mai, Thailand; ^2^Department of Biology, Faculty of Science, Chiang Mai University, Chiang Mai, Thailand; ^3^Genetics and Molecular Biology Unit, Faculty of Applied Sciences, University of Sri Jayewardenepura, Gangodawila, Sri Lanka; ^4^Academy of Science, The Royal Society of Thailand, Bangkok, Thailand

**Keywords:** 3 new species, China, molecular phylogeny, morphology, new host records, saprobic fungi, taxonomy, Thailand

## Abstract

Fungal taxonomy has a long history and changed significantly in the last few decades. Most recent studies have witnessed morphology combined with DNA-based molecular analyses as the main research tool for fungal species identification. During field surveys, some interesting *Didymosphaeriaceae* species were found from plant litter in China and Thailand. Morphology combined with phylogenetic analyses (Bayesian and maximum likelihood) of ITS, LSU, SSU, *tef1-*α, and *tub2* loci was used to identify fungal taxa. In this article, three new species and six new host records are described. The new species, *Montagnula acaciae, Paraconiothyrium zingiberacearum*, and *Paraphaeosphaeria brachiariae*, can be distinguished from other species of the respective genera based on their distinct size differences (ascomata, asci, and ascospores) and DNA sequence data. The new host records, *Montagnula jonesii, Paraconiothyrium fuckelii, Spegazzinia deightonii*, and *S. tessarthra* are reported from *Ficus benjamina, Dimocarpus longan, Hedychium coronarium*, and *Acacia auriculiformis* respectively, for the first time. Also, *Paraconiothyrium archidendri* and *P. brasiliense* are reported for the first time from *Magnolia* sp. in China. Moreover, *Paraconiothyrium rosae* is synonymized under *P. fuckelii* based on close phylogeny affinities and morphological characteristics. In-depth morphological descriptions, micrographs, and phylogenetic trees are provided to show the placement of new taxa.

## Introduction

Fungi are an essential and indispensable component of the ecosystem (Hawksworth, 2001; Gafforov, 2017; Hernandez-Restrepo et al., 2017; Purahong et al., 2017; Field and Pressel, 2018; Hyde et al., 2018a,b). They perform irreplaceable functions in the ecosystem such as nutrient cycling, responding to plant growth, maintaining plant diversity, and organic matter decomposition (Osono, 2017; Purahong et al., 2017; Li et al., 2018). Fungi are regarded as “key players” in the decomposition of litter due to their capability of secreting different kinds of enzymes (Promputtha et al., 2017; Tennakoon et al., 2021). The lignocellulose matrix of litter, which cannot be broken down by most species, is degraded by these enzymes (Romaní et al., 2006; Osono, 2007; Zhang et al., 2018; Tennakoon et al., 2021). Fungi have different lifestyles, such as biotrophs, endophytes, hemi-biotrophs, necrotrophs, and saprotrophs (Barelli et al., 2016; Hyde et al., 2018a; Tang et al., 2018; Tennakoon et al., 2021). They also differ greatly in terms of morphological characteristics, phylogeny characteristics, reproduction modes, and life cycles (Drinkwater et al., 2008; Purahong et al., 2017; Willis, 2018; Phookamsak et al., 2019). Therefore, identification of fungi is crucial to understanding their diversity and roles in the ecosystem. However, proper identification of fungal species is challenging. Despite challenges, mycologists have put considerable effort to identify fungal species worldwide (Lumbsch et al., 2011; Błaszkowski et al., 2013; Csata et al., 2013; Hyde et al., 2013; Giraldo et al., 2019).

*Didymosphaeriaceae*, one of the *Dothideomycetes* families, is an interesting taxonomic group of fungi in *Pleosporales* (Hyde et al., 2013; Hongsanan et al., 2020). Munk (1953) reported this family to contain *Didymosphaeria*. *Didymosphaeriaceae* members are morphologically diverse, and most of the sexual morphs have uni-septate, brown ascospores (Hongsanan et al., 2020). Asexual morph can be either coelomycetes (e.g., *Alloconiothyrium, Letendraea, Paraconiothyrium*, and *Paraphaeosphaeria*) or hyphomycetes (e.g., *Spegazzinia*) (Ariyawansa et al., 2014a,b; Thambugala et al., 2017; Hongsanan et al., 2020). The members of this family are cosmopolitan, and most of them have been recognized as saprobes. Meanwhile, some have been found as pathogens or endophytes in diverse plant substrates (e.g., leaves and twigs) in different ecosystems (e.g., marine, terrestrial, and mangroves) (Ariyawansa et al., 2013; Hyde et al., 2013; Liu et al., 2015; Wanasinghe et al., 2016; Hongsanan et al., 2020). Recently, numerous genera have been introduced in *Didymosphaeriaceae*. For instance, during the past 2 years period, four genera have been introduced, namely, *Cylindroaseptospora* (Jayasiri et al., 2019), *Neptunomyces* (Gonçalves et al., 2019), *Vicosamyces* (Phookamsak et al., 2019), and *Chromolaenicola* (Mapook et al., 2020). Hongsanan et al. (2020) added 32 genera in this family. Of them, some are highly diverse (e.g., *Didymosphaeria, Kalmusia, Paraphaeosphaeria, Pseudocamarosporium, Pseudopithomyces*, and *Spegazzinia*), and some genera have a few number of species (e.g., *Alloconiothyrium, Austropleospora, Barria, Bimuria, Cylindroaseptospora, Deniquelata, Didymocrea, Kalmusibambusa, Lineostroma, Vicosamyces*, and *Xenocamarosporium*) (Hongsanan et al., 2020).

As a part of the fungal diversity study, we have identified nine taxa from plant litter substrates (China and Thailand), which belong to the family *Didymosphaeriaceae*. Their taxonomic positions were established through morphology combined with phylogenetic analyses.

## Materials and methods

### Sample collection, morphological studies, and isolation

Plant litter (dead leaves and stems) samples were collected from China and Thailand. All the collected samples were examined under a stereo-microscope (AXIOSKOP 2 PLUS Series, Göttingen, Germany). Squash mount preparations were prepared to determine fungal microscopic features (e.g., asci, ascospores, conidia, and pseudoparaphyses). All the images were captured under an Axioskop 2 Plus (Göttingen, Germany) compound microscope equipped using a Canon Axiocam 506 color digital camera (Hanover, Germany). Lactoglycerol and nail polish were used to prepare the permanent slides. All the photo plates were prepared by Adobe Photoshop CS3 Extended version 10.0 software (Adobe Systems, USA), and measurements were taken by ZEN2 (blue edition).

### DNA extraction and polymerase chain reaction (PCR) amplification

The isolation process was used for the single spore isolation, as described by Senanayake et al. (2020). Potato dextrose agar (PDA) was used to transfer germinated spores, which were then incubated at 25°C. Following that, careful sub-culturing was carried out to obtain pure cultures. After 3 weeks, culture characteristics (on PDA) were observed. All the type specimens were deposited in the herbariums of Mae Fah Luang University (MFLU) and National Chiayi University (NCYU). The culture collections of Mae Fah Luang University (MFLUCC) and National Chiayi University (NCYUCC) were used to deposit the living cultures. Numbers for Faces of Fungi and Index Fungorum were used as mentioned in Jayasiri et al. (2015) and Index Fungorum (2022).

The fungal colonies growing on PDA (3 weeks) were used for genomic DNA extraction. Liquid nitrogen was used to grind the mycelium into a fine powder, and the DNA was extracted using the DNA extraction kit (E.Z.N.A Fungal DNA Mini Kit, D3390-02, Omega Bio-Tek) following the manufacturer's instructions. *Montagnula jonesii* (MFLU 18-0084) was subjected to direct DNA extraction using a DNA extraction kit from BioFlux^®^, Hangzhou, P.R. China, in accordance with the manufacturer's instructions. The DNA products were stored for a long period of time at −20 °C and retained at 4°C for DNA amplification. DNA was amplified by polymerase chain reaction (PCR) for obtaining the five genes: the large subunit (28S, LSU), small subunit (18S, SSU), internal transcribed spacers (ITS1-5.8S-ITS2), translation elongation factor 1-alpha gene (*tef1-*α), and β-tubulin (*tub2*). The LSU gene was amplified using the primers LR0R (5′-TCCTGAGGGAAACTTCG-3′) and LR5 (5′-ACCCGCTGAACTTAAGC-3′) (Vilgalys and Hester, 1990; Rehner and Samuels, 1994); the SSU gene was amplified using the primers NS1 (5′-GTAGTCATATGCTTGTCTC-3′) and NS4 (5′-CTTCCGTCAATTCCTTTAAG-3′) (White et al., 1990); nuclear ITS was amplified using the primers ITS5 (5′-GGAAGTAAAAGTCGTAACAAGG-3′) and ITS4 (5′-TCCTCCGCTTATTGATATGC-3′) (White et al., 1990); *tef1-*α gene was amplified using the primers EF1-983F (5′-GCYCCYGGHCAYCGTGAYTTYAT-3′) and EF1-2218R (5′-ATGACACCRACRGCRACRGTYTG-3′) (Rehner, 2001); and beta-tubulin (*tub2*) gene was amplified using the primers BT2a (5′-GGTAACCAAATCGGTGCTGCTTTC-3′) and BT2b (5′-ACCCTCAGTGTAGTGACCCTTGGC-3′) (Glass and Donaldson, 1995). Sterilized water (9.5 μl), 2 × Power Taq PCR MasterMix (Bioteke Co., China) (12.5 μl), each forward and reverse primers (1 μl), and DNA template (1 μl) were used for amplification reactions. The PCR thermal cycle program for ITS, LSU, SSU, *tef1-*α, and *tub2* was performed, as described by Conforto et al. (2019) and Tennakoon et al. (2020). PCR products were sent to Shanghai Sangon Biological Engineering Technology and Services Co., Ltd, China, for the purification and sequencing. All the obtained sequences were deposited in GenBank (Tables 1–4).

**Table 1:** GenBank and culture collection accession numbers of species included in this phylogenetic study (*Montagnula* tree).

**Species**	**Strain/voucher No**.	**GenBank accession No**.			
		**LSU**	**SSU**	**ITS**	***tef1-α***
*Karstenula rhodostoma*	CBS 690.94	GU301821	GU296154	–	GU349067
*K. rhodostoma*	CBS 691.94	AB807531	AB797241	LC014559	AB808506
***Montagnula acaciae***	**MFLUCC 18-1636**	**ON117298**	**ON117267**	**ON117280**	**ON158093**
***M. acaciae***	**NCYUCC 19-0087**	**ON117299**	**ON117268**	**ON117281**	**ON158094**
*M. aloes*	CPC 19671	JX069847	–	JX069863	–
*M. appendiculata*	CBS 109027	AY772016	–	DQ435529	–
*M. bellevaliae*	MFLUCC 14-0924	KT443902	KT443904	KT443906	–
*M. camporesii*	MFLUCC 16-1369	MN401742	MN401744	MN401746	MN397908
*M. chiangraiensis*	MFLUCC 17-1420	MT214443	MT214397	MT214349	–
*M. chromolaenae*	MFLUCC 17-1435	MT214444	MT214398	MT214350	–
*M. chromolaenicola*	MFLUCC 17-1469	MT214445	MT214399	MT214351	MT235773
*M. cirsii*	MFLUCC 13-0680	KX274249	KX274255	KX274242	KX284707
*M. donacina*	HFG07004	MF183940	–	MF967419	–
*M. donacina*	HVVV01	KJ628377	KJ628376	KJ628375	–
*M. graminicola*	MFLUCC 13-0352	KM658315	KM658316	KM658314	–
*M. jonesii*	MFLUCC 16-1448	KY273276	KY313618	KY313619	KY313620
***M. jonesii***	**MFLU 18-0084**	**ON117300**	**ON117269**	**ON117282**	**ON158095**
*M. krabiensis*	MFLUCC 16-0250	MH260303	MH260343	MH275070	MH412776
*M. opulenta*	UTHSC: DI16-208	LN907351	–	LT796834	LT797074
*M. puerensis*	KUMCC 20-0225	MW575866	MW575864	MW567739	MW573959
*M. puerensis*	KUMCC 20-0331	MW575867	MW575865	MW567740	MW573960
*M. saikhuensis*	MFLUCC 16-0315	KU743210	KU743211	KU743209	–
*M. scabiosae*	MFLUCC 14-0954	KT443903	KT443905	KT443907	–
*M. thailandica*	MFLUCC 17-1508	MT214446	MT214400	MT214352	MT235774
*M. rhodophaea*	CBS 616.86	–	GU205249	–	–

The newly generated sequences are shown in bold black.

**Table 2:** GenBank and culture collection accession numbers of species included in this phylogenetic study (*Paraconiothyrium* tree).

**Species**	**Strain/voucher No**.	**GenBank accession No**.		
		**LSU**	**ITS**	***tub2***
*Paraconiothyrium ajrekarii*	NFCCI 4810	MT372905	MT372906	MT394161
*P. archidendri*	CBS 168.77	MH872813	MH861045	JX496388
*P. archidendri*	964-SAB SA1 3	–	MT820342	–
*P. archidendri*	C321	MK347974	MK347757	–
*P. archidendri*	1–3–10–2–1–4	–	KX065269	–
*P. archidendri*	NNIBRFG116	–	KY327413	–
*P. archidendri*	NNIBRFG99	–	KY327412	–
*P. archidendri*	NNIBRFG29	–	KY327411	–
***P. archidendri***	**MFLUCC 19-0043**	**ON117302**	**ON117284**	–
*P. babiogorense*	CBS 128292	MH876291	MH864845	–
*P. brasiliense*	CBS 115.92	JX496135	JX496022	JX496361
*P. brasiliense*	CBS 395.87	JX496196	JX496083	JX496422
*P. brasiliense*	CBS 122320	JX496146	JX496033	JX496372
*P. brasiliense*	CBS 122851	JX496149	JX496036	JX496375
***P. brasiliense***	**MFLUCC 19-0040**	**ON117301**	**ON117283**	–
*P. camelliae*	NTUCC 18-096	MT071269	MT112293	MT308623
*P. cyclothyrioides*	CBS 972.95	JX496232	JX496119	JX496458
*P. cyclothyrioides*	CBS 432.75	JX496201	JX496088	JX496427
*P. cyclothyrioides*	SN 3169-19	–	MN416682	–
*P. cyclothyrioides*	R-4779	JQ681304	JQ681303	–
*P. cyclothyrioides*	NNIBRFG3266	MW237682	–	–
*P. cyclothyrioides*	NNIBRFG3255	MW237679	–	–
*P. cyclothyrioides*	EXF-14614	–	MT280696	–
*P. cyclothyrioides*	NFCCI 4387	MN241143	MN242780	–
*P. estuarinum*	CBS 109850	JX496129	JX496016	JX496355
*P. fici*	NI145	–	–	–
*P. fuckelii*	JZB320001	MN519513	MN495986	MN508193
*P. fuckelii*	MFLUCC 13-0073	–	–	–
*P. fuckelii*	JZB320002	MN519514	MN495987	MN508194
*P. fuckelii*	JZB320003	MN519515	MN495988	MN508195
*P. fuckelii*	JZB320004	MN519516	MN495989	MN508196
*P. fuckelii*	CBS 584.69	–	JX496211	JX496437
*P. fuckelii*	CVG970	–	MZ712975	
*P. fuckelii*	CBS 508.94	JX496209	JX496096	JX496435
*P. fuckelii*	CBS 653.85	JX496217	JX496104	JX496443
*P. fuckelii*	CBS 764.71B	JX496225	JX496112	JX496451
***P. fuckelii***	**MFLUCC 19-0067**	**ON117305**	**ON117287**	–
*P. fuckelii*	CBS 797.95	JX496226	JX496113	JX496452
*P. fuscomaculans*	CBS 116.16	MH866170	MH854649	–
*P. hakeae*	CBS 142521	KY979809	KY979754	KY979920
*P. iridis*	CBS:146036	MT223919	MT223827	MT223743
*P. lini*	CBS 253.92	GU238093	–	KT266268
*P. lycopodinum*	CBS 134705	MH877564	–	–
*P. maculicutis*	CBS 101461	EU754200	–	–
*P. magnoliae*	MFLUCC 10-0278	KJ939283	KJ939280	–
*P. nelloi*	MFLU 14-0813	KP711365	KP711360	–
*P. polonense*	CBS 134153	KF700360	–	–
*P. rosae*	MFLU 15-1115	MG829041	MG828932	–
*P. thysanolaenae*	MFLUCC 10-0550	KP744496	KP744453	–
*P. tiliae*	CBS 265.94	EU754139	–	–
***P. zingiberacearum***	**MFLUCC 18-0559**	**ON117303**	**ON117285**	**ON158098**
***P. zingiberacearum***	**NCYUCC 19-0230**	**ON117304**	**ON117286**	**ON158099**
*Tremateia arundicola*	MFLU 16-1275	KX274248	KX274241	–
*Tremateia guiyangensis*	GZAAS01	KX274247	KX274240	–

The newly generated sequences are shown in bold black.

**Table 3:** GenBank and culture collection accession numbers of species included in this phylogenetic study (*Paraphaeosphaeria tree*).

**Species**	**Strain/Voucher No**.	**GenBank Accession no**.			
		**LSU**	**SSU**	**ITS**	***tub2***
*Paraconiothyrium fuckelii*	JZB320002	MN519514	–	MN495987	MN508194
*P. fuckelii*	JZB320001	MN519513	–	MN495986	MN508193
*P. fuckelii*	JZB320003	MN519515	–	MN495988	MN508195
*Paraphaeosphaeria* sp.	CBS 101464	JX496125	–	JX496012	JX496351
*Para. angularis*	CBS 167.70	MH871317	–	JX496047	JX496386
*Para. arecacearum*	CBS 158.75	JX496156	–	JX496043	JX496382
***Para. brachiariae***	**MFLU 19-2799**	**ON117306**	**ON117270**	**ON117288**	**ON158100**
***Para. brachiariae***	**NCYU 19-0058**	**ON117307**	**ON117271**	**ON117289**	**ON158101**
*Para. cameliae*	NTUCC 18-095-1	MT071267	MT071218	MT112291	MT308621
*Para. graminicola*	MFLUCC 15-0450	KX954398	KX986342	KX965729	KY197981
*Para. hydei*	HNNU0523	MK329032	–	OL774782	MK336140
*Para. michotii*	MFLUCC 15-0041	MG829042	MG829148	MG828933	–
*Para. michotii*	MFLUCC 15-0043	MG829043	MG829149	MG828934	–
*Para. michotii*	MFLUCC 13-0349	KJ939282	KJ939285	KJ939279	–
*Para. minitans*	CBS 111750	JX496130	–	JX496017	JX496356
*Para. neglecta*	CBS 124078	MH874872	–	MH863348	JX496378
*Para. neglecta*	CBS 119637	JX496138	–	JX496025	JX496364
*Para. parmeliae*	CBS 131728	KP170722	–	KP170654	KP170703
*Para. pilleata*	CBS 102207	JX496126	–	JX496013	JX496352
*Para. rosae*	MFLUCC 17-2547	MG829044	MG829150	MG828935	–
*Para. rosicola*	MFLU 18-0108	MG829047	MG829153	MG828938	–
*Para. sardoa*	CBS 501.71	MH872003	–	MH860235	JX496433
*Para. spartii*	MFLU 14-C0810	KP711362	KP711367	KP711357	–
*Para. sporulosa*	CBS 391.86	JX496195	–	JX496082	JX496421
*Para. sporulosa*	CBS 105.76	JX496127	–	JX496014	JX496353
*Para. verruculosa*	CBS 263.85	MH873567	–	MH861879	JX496398
*Para. viciae*	MFLU 15-1231	KY397947	KY397948	KY379969	–
*Para. viridescens*	CBS 854.73	MH872545	–	JX496085	JX496424
*P. xanthorrhoeae*	CBS 142164	KY979793	–	KY979738	KY979909

The newly generated sequences are shown in bold black.

**Table 4:** GenBank and culture collection accession numbers of species included in this phylogenetic study (*Spegazzinia* tree).

**Species**	**Strain/voucher No**.	**GenBank accession No**.			
		**LSU**	**SSU**	**ITS**	***tef1-α***
*Laburnicola muriformis*	MFLUCC 16-0290	KU743198	KU743199	KU743197	–
*L. muriformis*	MFLUCC 14-0921	KU743201	KU743202	KU743200	–
*Spegazzinia* sp.	yone 279	AB807583	AB797293	–	AB808559
*Spegazzinia* sp.	CL115	AY234948	–	–	–
*S. bromeliacearum*	URM 8084	MK809513	–	MK804501	–
*S. cameliae*	CMU 328	MH734521	MH734523	MH734522	MH734524
*S. deightonii*	yone 212	AB807582	AB797292	–	AB808558
*S. deightonii*	MFLUCC 20-0002	MN956772	MN956770	MN956768	MN927133
*S. deightonii*	yone 66	AB807581	AB797291	–	AB808557
*S. deightonii*	yone 66	AB807581	AB797291	–	–
***S. deightonii***	**MFLUCC 18-1625**	**ON117309**	**ON117273**	**ON117291**	**ON158097**
*S. intermedia*	CBS 249.89	MH873861	–	MH862171	–
*S. lobulata*	CBS 361.58	MH869344	–	MH857812	–
*S. musae*	MFLUCC 20-0001	MN930514	MN930513	MN930512	MN927132
*S. neosundara*	MFLUCC 15–0456	KX954397	KX986341	KX965728	–
*S. neosundara*	MFLUCC 13-0211	MH040812	MH040811	MH040810	MH055460
*S. radermacherae*	MFLUCC 17-2285	NG_066308	MK347848	NR_163331	MK360088
*S. tessarthra*	SH 287	AB807584	AB797294	–	AB808560
*S. tessarthra*	NRRL 54913	–	–	JQ673429	–
*S. tessarthra*	ASV319	–	–	MN898233	–
*S. tessarthra*	MFLUCC 17-2249	MH071197	MH071192	MH071193	–
***S. tessarthra***	**MFLUCC 18-1624**	**ON117308**	**ON117272**	**ON117290**	**ON158096**
*S. tessarthra*	12H0104	–	–	KT385776	–

The newly generated sequences are shown in bold black.

### Phylogenetic analyses

A combined gene dataset of ITS, LSU, SSU, *tef1-*α, and *tub2* was used for the phylogenetic analyses. The newly attained sequences were initially subjected to BLASTn searches in GenBank (http://www.ncbi.nlm.nih.gov/) to identify the taxa that shared the most similarities with our strains. The additional sequences included in the analysis were from previous publications (Phookamsak et al., 2019; Samarakoon et al., 2020; Boonmee et al., 2021; Tennakoon et al., 2021). MAFFT v.7 web server (http://mafft.cbrc.jp/alignment/server) was used to make multiple alignments (Katoh and Standley, 2013). The alignment was corrected manually using BioEdit v.7.0.5.2 (Hall, 1999), where necessary.

The CIPRES Science Gateway platform (Miller et al., 2010) was used to create maximum likelihood trees using the GTR + I + G model of evolution and RAxML-HPC2 on XSEDE (8.2.8) (Stamatakis et al., 2008; Stamatakis, 2014). MrModeltest v.3.7 (Posada and Crandall, 1998) was carried out to check the evolutionary models for phylogenetic analyses under the Akaike Information Criterion (AIC). Model test results revealed “GTR + I + G” as the best-fit model for each locus for Bayesian and maximum likelihood analyses. MrBayes v. 3.1.2 (Huelsenbeck and Ronquist, 2001) was used for Bayesian analysis (Rannala and Yang, 1996; Zhaxybayeva and Gogarten, 2002). The number of generations used in Bayesian analysis were 1,000,000 to 5,000,000, and trees were sampled every 100th or 1,000th generations. The FigTree v1.4.0 (Rambaut, 2012) program was used to view the phylogenetic trees and was reorganized by using Microsoft PowerPoint (2010). All the final alignments were deposited in TreeBASE (http://www.treebase.org/) (Submission ID: 29615).

## Results

### Phylogenetic and taxonomic results of *Montagnula*

#### *Montagnula* Berl

Notes: Berlese (1896) established this genus to include *M*. *infernalis* as the generic type. *Montagnula* members mostly have globose, spherical, immersed, or semi-immersed ascomata, clavate to cylindrical asci, and multi-septate, fusoid, or ellipsoid ascospores (Ariyawansa et al., 2014a; Pitt et al., 2014). *Montagnula* species serves a crucial role in the environment as saprobes, which generally grow on the wood and bark of dead plants but also rarely on dead leaves (Hongsanan et al., 2020; Mapook et al., 2020). This genus has a vast range of species all around the world. In addition, given the number of new species revealed through recent investigations, *Montagnula* appears to be phylogenetically diverse as well (Mapook et al., 2020). Up to date, 39 accepted *Montagnula* species are listed in Index Fungorum (2022). In this study, we introduce *Montagnula acaciae* as a new species and new host record of *M. jonesii* from *Ficus benjamina*.

**Figure 1:**
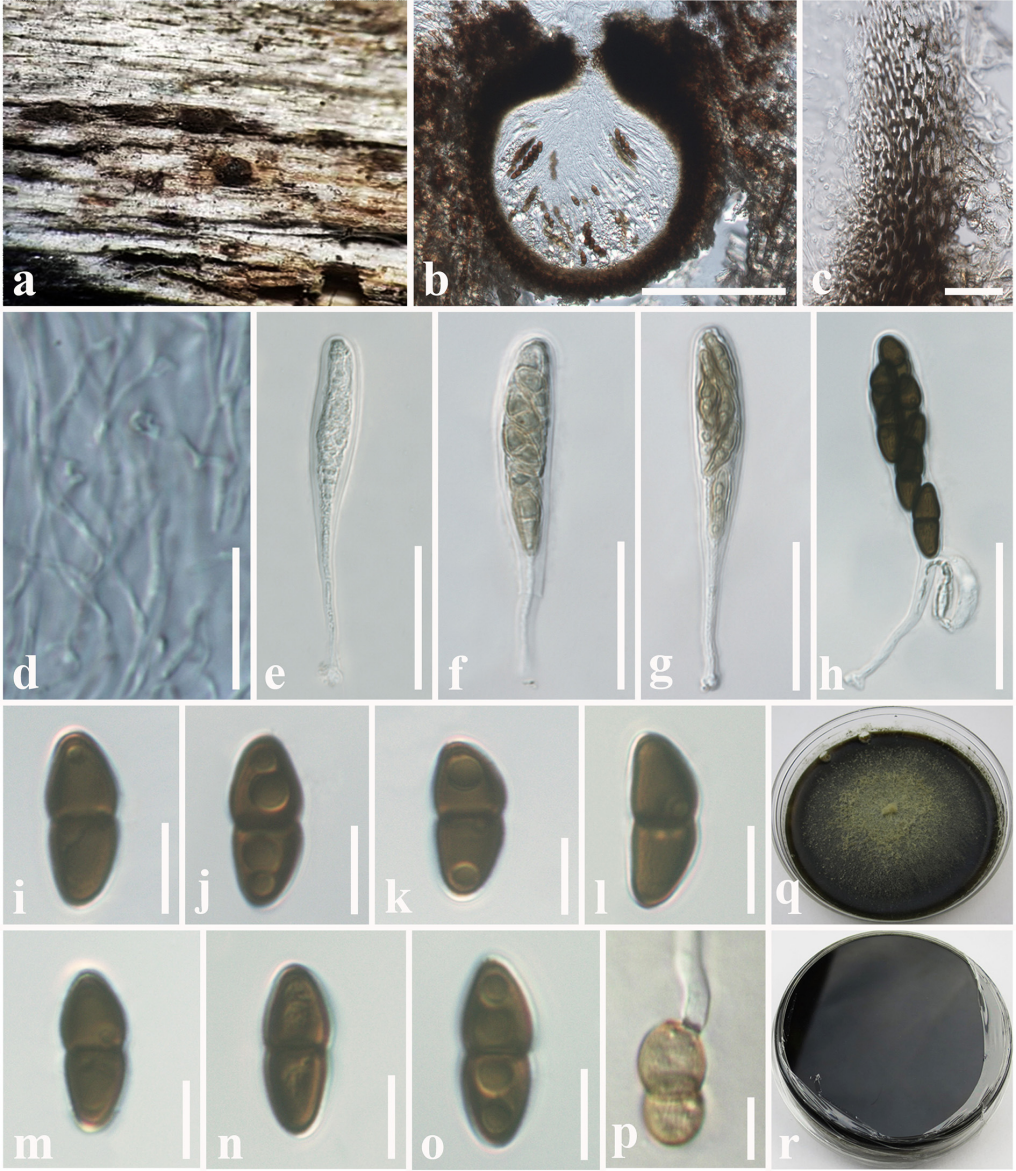
*Montagnula acaciae* (MFLU 18-2569, holotype). **(a)** Ascomata on a dead stem of *Acacia auriculiformis*. **(b)** Vertical section through an ascoma. **(c)** Peridium. **(d)** Pseudoparaphyses. **(e–h)** Immature and mature asci. **(i–o)** Ascospores. **(p)** Germinating ascospore. **(q)** Upper side of the culture. **(r)** Lower side of the culture. Scale bars: **(b)** = 100 μm, **(c)** = 8 μm, **(d–h)** = 30 μm, **(i–p)** = 5 μm.

***Montagnula acaciae*** Tennakoon and S. Lumyong, sp. nov. (Figure 1).

Etymology: Name reflects the host genus *Acacia*.

Index Fungorum Number: IF559599.

Facesoffungi number: FoF 10801.

Holotype: MFLU 18-2569.

*Saprobic* on dead stem of *Acacia auriculiformis* A. Cunn. (*Fabaceae*). **Sexual morph**: *Ascomata* 140–180 μm high × 150–200 μm diam. ($\bar{x}$ = 160 × 175 μm, *n* = 10), solitary or scattered, coriaceous, immersed to semi-immersed, partly erumpent, unilocular, globose to obpyriform, brown to dark brown, ostiolate, with minute papilla, filled with hyaline cells. *Peridium* 10–20 μm wide, outer layer comprising light brown to dark brown, thick-walled, cells of *textura angularis*, inner layer comprising hyaline, flattened, thin-walled cells of *textura angularis*. *Hamathecium* comprising 1.3–2.4 μm wide, cylindrical to filiform, branched, septate pseudoparaphyses. *Asci* 50–90 × 8–10 μm ($\bar{x}$ = 72 × 9 μm, *n* = 20), 8-spored, bitunicate, fissitunicate, clavate to cylindrical, with a long pedicel, slightly curved, rounded at the apex and with a shallow ocular chamber. *Ascospores* 10–15 × 4–7 μm ($\bar{x}$ = 12 × 6 μm, *n* = 30), overlapping, 1–2-seriate, fusiform to ellipsoid, initially hyaline or pale brown, becoming light brown to dark brown, 1-septate, constricted at the septum, upper cell slightly wider, tapering toward ends, slightly curved. **Asexual morph**: Undetermined.

Culture characteristics: *Colonies* growing on PDA, 15–20 mm diam. after 3 weeks at 25°C, colonies circular, dense, flattened, surface slightly rough, entire edge, velvety; from above: dark brown at margin, gray to slightly greenish at center; reverse: dark brown to black at the margin and center, mycelium greenish to greenish gray.

Material examined: Thailand, Chiang Mai, on dead stem of *Acacia auriculiformis* (*Fabaceae*), 25 March 2016, D.S. Tennakoon, TD001A (MFLU 18-2569, holotype); ex-type living culture, MFLUCC 18-1636. *ibid*. 28 March 2016, TD001B (NCYU 19-0255, Paratype); ex-paratype living culture, NCYUCC 19-0087.

**Figure 2:**
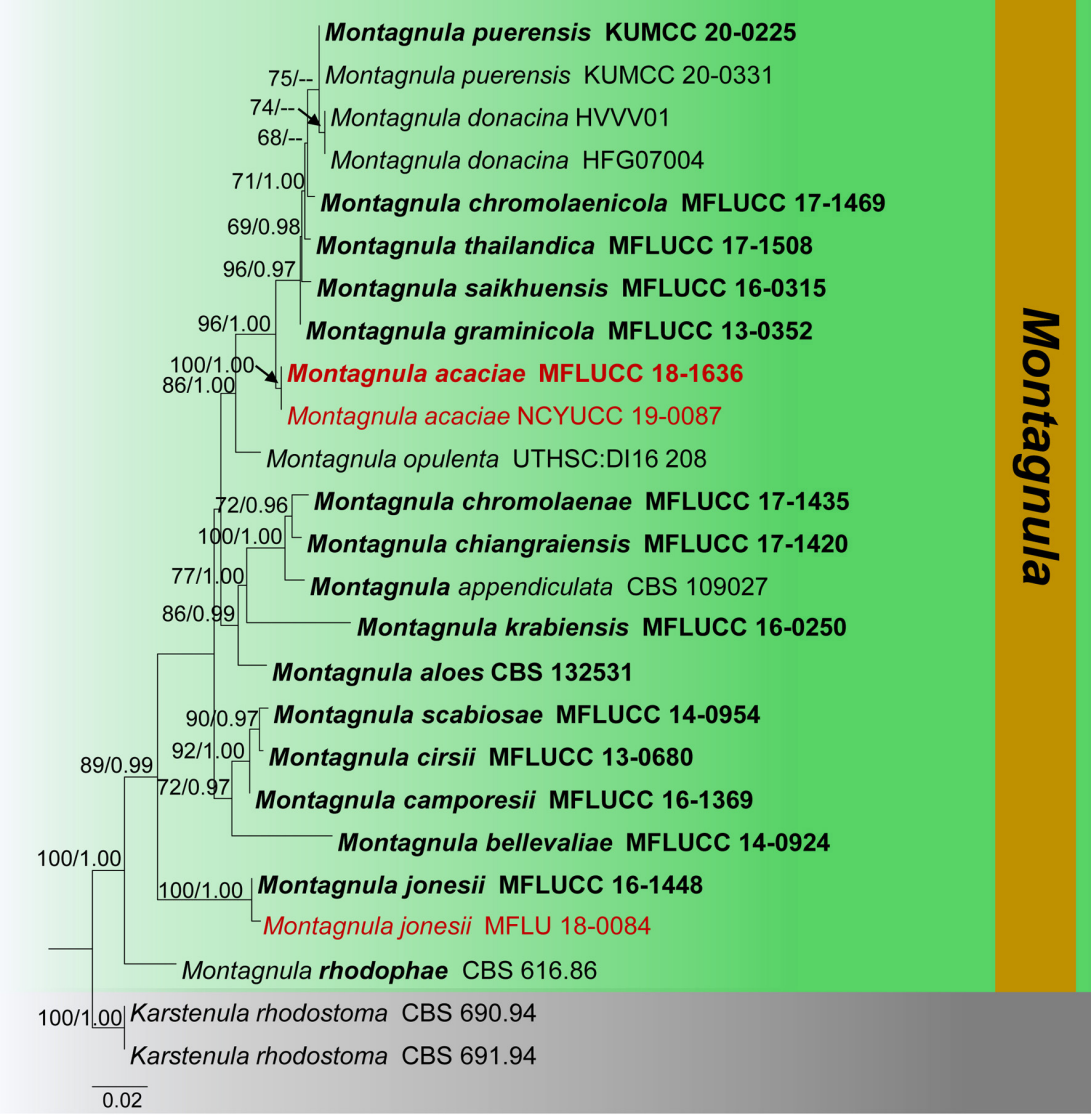
Phylogram generated from maximum likelihood analysis is based on combined ITS, LSU, and SSU sequence data (Final likelihood value of −9216.328354). The tree is rooted with *Karstenula rhodostoma* (CBS 690.94 and CBS 691.94). The ex-type strains are indicated in bold, and the new isolates are indicated in red. Bootstrap support values ≥65% from the maximum likelihood (ML) and Bayesian posterior probabilities (PP) values ≥0.90 are given above the nodes.

Notes: The morphological characteristics of *Montagnula acaciae* agree well with those described for *Montagnula* species (immersed to semi-immersed or erumpent ascomata; clavate to cylindrical, long pedicellate asci; and light brown to dark brown, 1-septate ascospores) (Hyde et al., 2013; Hongsanan et al., 2020; Mapook et al., 2020). The generated phylogeny (LSU, SSU, ITS, and *tef1-*α) shows that *M. acaciae* forms a distinct sister lineage to the clade formed by *M*. *chromolaenicola, M*. *donacina, M*. *graminicola, M*. *puerensis, M*. *saikhuensis*, and *M*. *thailandica* with strong statistical support (96% ML, 1.00 BYPP, Figure 2). Morphologically, *M. acaciae* differs from *M*. *saikhuensis* and *M*. *puerensis* in having smaller ascomata (140–180 × 150–200 μm), whereas *M*. *saikhuensis* and *M*. *puerensis* have larger ascomata (400–450 × 400–500 and 300–600 × 230–380 μm). *Montagnula donacina* differs from *M*. *acaciae* in having carbonaceous ascostromata with a flat bottom (Pitt et al., 2014). In addition, *M. graminicola* can be differentiated from *M*. *acaciae* in having verrucous ascospores, not constricted in the middle, and surrounded by obvious sheaths (Liu et al., 2015). Interestingly, we found that the morphological characteristics of *M. acaciae* share similarities with *M*. *thailandica*, but they are phylogenetically distinct (Figure 2). We compared the base pair differences of the ITS (+5.8S) gene region of *M. acaciae* and *M*. *thailandica*, and there were 12 base pair differences (2.18%) across 550 nucleotides. In addition, there were 22 base pair differences (2.51%) across 880 nucleotides of *tef1-*α gene region as well. The main morphology differences of *Montagnula* species are provided in Table 5.

**Table 5:** Synopsis of *Montagnula* species treated in this study.

***Montagnula* Species**	**Size (**μ**m)**			**Septa**	**Host**	**References**
	**Ascomata**	**Asci**	**Ascospores**			
***M. acaciae***	**140–180** **× 150–200**	**50–90** **× 8–10**	**10–15** **× 4–7**	**1**	***Acacia auriculiformis***	**This study**
*M. aloes*	450 diam.	110–250 × 20–30	33–36 × 13–14	3	*Aloe* sp.	Crous et al., 2012
*M. appendiculata*	100–200	–	12–15 × 4–5	1	*Zea mays*	Aptroot, 2004
*M. bellevaliae*	100–120 × 150–175	70–100 × 9–12	15–18 × 5–6	2	*Bellevalia romana*	Hongsanan et al., 2015
*M. camporesii*	200–250 × 300–350	80–120 × 10–15	18–25 × 5–8	3	*Dipsacus* sp.	Hyde et al., 2020b
*M. chiangraiensis*	150–220 × 200–230	60–75 × 8–11	11–15 × 4–6	1	*Chromolaena odorata*	Mapook et al., 2020
*M. chromolaenae*	170–175 × 170–190	85–105 × 9–15	15–16.5 × 5–6	1	*Chromolaena odorata*	Mapook et al., 2020
*M. chromolaenicola*	300–320 × 215–310	80–100 × 10–13	15–17 × 5–6.5	1	*Chromolaena odorata*	Mapook et al., 2020
*M. cirsii*	385–415 × 510–525	84.5–119.5 × 10.5–13.5	18–23.5 × 6.5–9.5	3	*Cirsium* sp.	Hyde et al., 2016
*M. donacina*	–	–	12–17 × 4–6.5	1	*Arundo donax*	Aptroot, 1995
*M. graminicola*	37–117.22	50–132 × 8–13	9.8–13 × 3.8–5.5	1	Grass sp.	Liu et al., 2015
*M. jonesii*	325–350 × 300–325	72–95 × 9–13	14–16 × 5–6	3	*Fagus sylvatica*	Tennakoon et al., 2016
***M. jonesii***	**150–200** **× 175–275**	**60–72** **× 8–10**	**13.5–16** **× 4.5–5.5**	**3**	***Ficus benjamina***	**This study**
*M. krabiensis*	140–160 × 150–170	70–125 × 15–20	55–32 × 6–7	1	*Pandanus* sp.	Tibpromma et al., 2018
*M. opulenta*	400–1200	–	19–25 × 9–13	1	*Opuntia* sp.	Aptroot, 1995
*M. puerensis*	300–600 × 230–380	92 × 11	14 × 6	1	*Acer* sp.	Du et al., 2021
*M. saikhuensis*	400–450 × 400–500	70–100 × 10–12	12–16 × 4–6	1	*Citrus* sp.	Wanasinghe et al., 2016
*M. scabiosae*	300–320 × 300–360	110–130 × 14–20	20–23 × 7–9	3	*Scabiosa* sp.	Hongsanan et al., 2015
*M. thailandica*	405–415 × 330–350	80–100 × 9–15	14–17 × 4.5–7.5	1	*Chromolaena odorata*	Mapook et al., 2020

**Figure 3:**
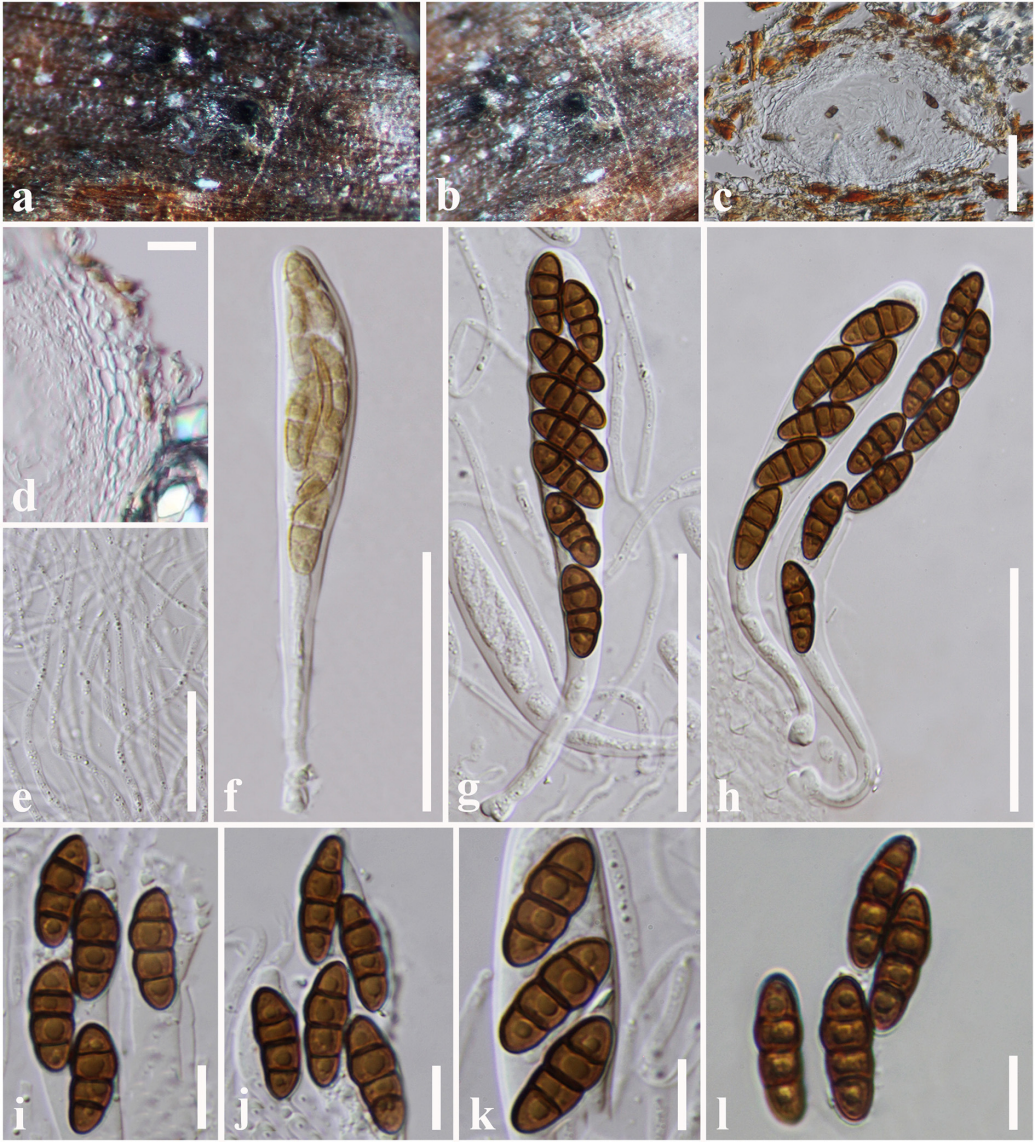
*Montagnula jonesii* (MFLU 18-0084, new host record). **(a,b)** Ascomata on a dead stem of *Ficus benjamina*. **(c)** Vertical section through an ascoma. **(d)** Peridium. **(e)** Pseudoparaphyses. **(f–h)** Asci. **(i–l)** Ascospores. Scale bars: **(c)** = 50 μm, **(d)** = 10 μm, **(e–h)** = 30 μm, **(i–l)** = 8 μm.

***Montagnula jonesii*** Tennakoon, Wanas., Phook. and K.D. Hyde, Mycosphere 7: 1350 (2016) (Figure 3).

Index Fungorum Number: IF552577.

Facesoffungi number: FoF 02719.

*Saprobic* on dead stem of *Ficus benjamina* L. (*Moraceae*). **Sexual morph**: *Ascomata* 150–200 μm high × 175–275 μm diam. ($\bar{x}$ = 175 × 210 μm, *n* = 10), scattered to clustered, solitary, immersed to semi-immersed, erumpent, globose to sub-globose, glabrous, dark brown to black, uniloculate, ostiolate. *Peridium* 10–20 μm wide, thin-walled with equal thickness, composed of several layers of hyaline, pseudoparenchymatous cells, arranged in a *textura angularis*. *Hamathecium* comprising 1.5–2.5 μm wide, cylindrical to filiform, cellular pseudoparaphyses, septate, not constricted at the septum, anastomosing at the apex, embedded in a gelatinous matrix. *Asci* 60–72 × 8–10 μm ($\bar{x}$ = 65 × 9 μm, *n* = 20), bitunicate, 8-spored, clavate, apically rounded, with a long pedicel, with an ocular chamber. *Ascospores* 13.5–16 × 4.5–5.5 μm ($\bar{x}$ = 15 × 5 μm), overlapping 1–2-seriate, ellipsoidal to fusiform with rounded ends, hyaline or pale brown when immature, brown to reddish-brown when mature, mostly 1-septate at immature, 3-septate at mature, constricted at the septa, slightly curved, second cell enlarge from apex, guttulate, smooth-walled. **Asexual morph**: Undetermined.

Known hosts: *Fagus sylvatica* and *Ficus benjamina* (Tennakoon et al., 2016; This study).

Known distribution: Italy and China (Tennakoon et al., 2016; This study).

Material examined: China, Taiwan region, Chiayi, Dahu Forest, on a dead stem of *Ficus benjamina* (*Moraceae*), 26 August 2018, D.S. Tennakoon, D008 (MFLU 18-0084).

**Figure 4:**
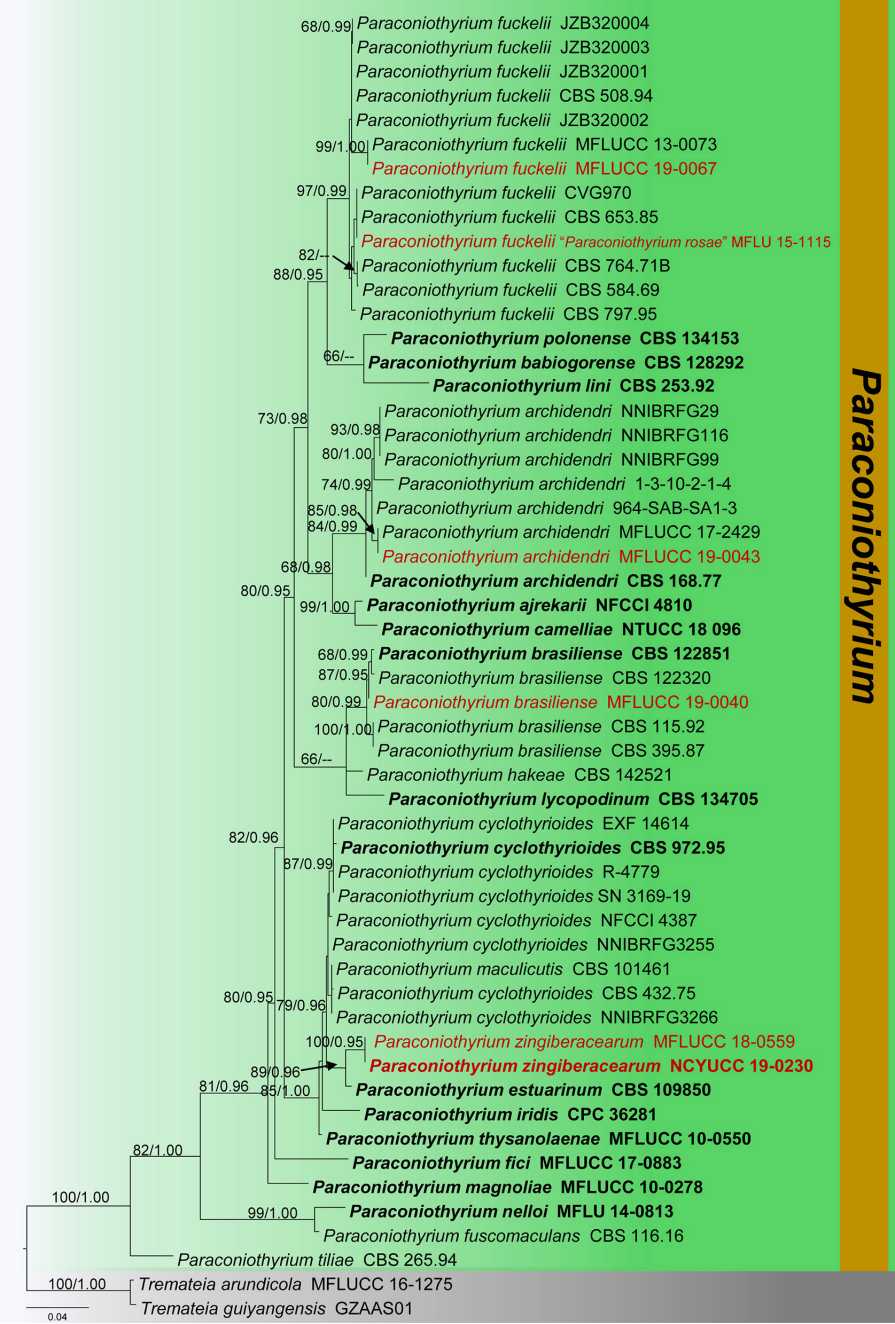
Phylogram generated from maximum likelihood analysis is based on combined ITS, LSU, and *tub2* sequence data (final likelihood value of −8278.609544). The tree is rooted with *Tremateia arundicola* and *T*. *guiyangensis* (MFLUCC 16-1275 and GZAAS01). The ex-type strains are indicated in bold, and the new isolates are indicated in red. Bootstrap support values ≥65% of maximum likelihood (ML) and Bayesian posterior probabilities (PP) values ≥0.90 are given above the nodes.

Notes: Tennakoon et al. (2016) introduced *Montagnula jonesii* from *Fagus sylvatica* in Italy. The morphological characteristics of our collection (MFLU 18-0084) resemble *M. jonesii* (MFLU 16-1363) in having globose to sub-globose, immersed to semi-immersed, erumpent ascomata; clavate, apically rounded, long pedicellate asci; and brown to reddish-brown, ellipsoidal to fusiform, 3-septate ascospores with enlarge second cell from apex (Tennakoon et al., 2016). Our phylogeny also shows that our collection cluster with *M. jonesii* (MFLU 16-1363), and it is supported by phylogenetic data (100% ML, 1.00 BYPP, Figure 4). We therefore consider MFLU 18-0084 as a new host record of *M. jonesii* from *Ficus benjamina*.

### Phylogenetic and taxonomic results of *Paraconiothyrium*

#### *Paraconiothyrium* Verkley

Notes: *Paraconiothyrium* is a diverse genus, which was introduced by Verkley et al. (2004). Members of this genus play a crucial function as saprobes on dead plants, particularly dead wood, and occasionally on dead leaves (Verkley et al., 2004; Hyde et al., 2013; Ariyawansa et al., 2014b; Hongsanan et al., 2020; Boonmee et al., 2021). In addition, since the species has been found in both temperate and tropical countries (e.g., Australia, China, India, Iran, Laos, Myanmar, Poland, Thailand, the United States, and Uzbekistan), *Paraconiothyrium* appears to have a global distribution (Verkley et al., 2004, 2014; Budziszewska et al., 2011; Ariyawansa et al., 2014b, 2020; Crous et al., 2017; Gafforov, 2017; Hongsanan et al., 2020; Boonmee et al., 2021). *Paraconiothyrium* has diverse morphology characteristics, such as eustromatic to pycnidial conidiomata, phialidic or annelidic conidiogenous cells, and hyaline to brown conidia (Verkley et al., 2004, 2014). Currently, 19 *Paraconiothyrium* species are accepted in Index Fungorum (2022). In this study, we introduce *Paraconiothyrium zingiberacearum* as a new species, and *P*. *archidendri, P*. *brasiliense* and *P*. *fuckelii* as new host records. In addition, *P*. *rosae* was synonymized under *P*. *fuckelii* based on strong morphological similarities and phylogeny data.

**Figure 5:**
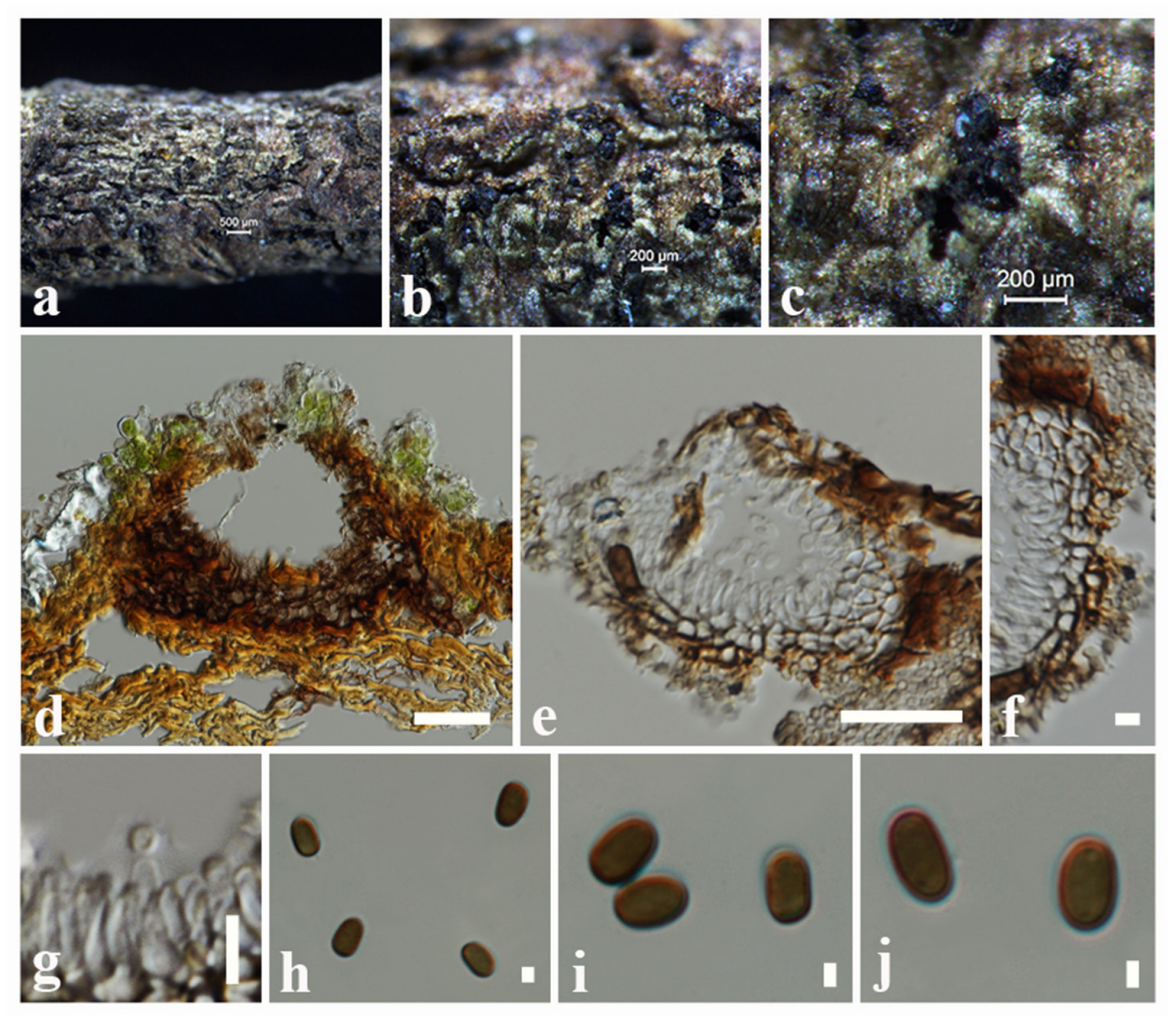
*Paraconiothyrium archidendri* (MFLU 18-2653, new host record): **(a)** Conidiomata on a dead twig of *Magnolia* sp. **(b,c)** Close-up of conidiomata on the host. **(d,e)** Sections through conidiomata. **(f)** Conidiomatal wall. **(g)** Conidiogenous cells with developing conidia. **(h–j)** Conidia. Scale bars: **(a)** = 500 μm, **(b,c)** = 200 μm, **(d,e)** = 50 μm, **(f,g)** = 10 μm, **(h–j)** = 2 μm.

***Paraconiothyrium archidendri*** Verkley, Göker and Stielow, Persoonia 32: 37 (2014) (Figure 5).

Index Fungorum Number: IF800761.

Facesoffungi number: FoF 05243.

*Saprobic* on dead twigs of *Magnolia* sp. (*Magnoliaceae*). **Sexual morph**: Undetermined. **Asexual morph**: Coelomycetous. *Conidiomata* 90–120 μm high, 100–140 μm diam. ($\bar{x}$ = 100 × 125 μm, *n* = 10), pycnidial, superficial or semi-immersed, dark brown to black, solitary to aggregated, globose to sub-globose. *Conidiomatal wall* 19–23 μm wide, 2–3 cell layers, composed of brown, cells of *textura angularis* with relatively thick-walled. *Conidiophores* reduced to conidiogenous cells. *Conidiogenous cells* 5–7 × 3–4 μm ($\bar{x}$ = 6 × 3.5 μm, *n* = 20), discrete, globose to doliiform, holoblastic, hyaline. *Conidia* 6–7 × 3–4 ($\bar{x}$ = 6.5 × 3.5 μm, *n* = 40) μm, variable in shape, ellipsoid, rarely obovoid, ends rounded, sub-globose or sometimes one end more or less blunt, aseptate, initially hyaline, becoming light brown at maturity, smooth-walled.

Culture characteristics: *Colonies* growing on PDA, 23 mm diam. after 1 week at 25°C, colonies circular, dense, surface smooth, undulate edge, cottony; from above: pale brown; reverse: brown.

Known hosts: *Leucaena* sp., *Pithecellobium bigeminum*, and *Magnolia* sp. (Verkley et al., 2014; Jayasiri et al., 2015; Goh et al., 2016; this study).

Known distribution: China, Korea, and Myanmar (Verkley et al., 2014; Jayasiri et al., 2015; Goh et al., 2016; this study).

Material examined: China, Xishuangbanna, on dead twigs of *Magnolia* sp. (*Magnoliaceae*), 27 March 2017, N.I. de Silva, NI275 (MFLU 18-2653), living culture, MFLUCC 19-0043.

Notes: Verkley et al. (2014) described *Paraconiothyrium archidendri*, which was associated with leaf spots of *Pithecellobium bigeminum* in Myanmar. Phylogenetic analyses show that a new strain (MFLUCC 19-0043) clustered with other strains of *P*. *archidendri*, in particular close to the isolate MFLUCC 17-2429 with 85% ML and 0.98 BYPP statistical support (Figure 4). The new collection (MFLU 18-2653) morphologically fits with the description of *P*. *archidendri* (CBS 168.77 and MFLUCC 17-2429) in having globose to sub-globose pycnidia, globose to doliiform conidiogenous cells and olivaceous-brown, sub-globose or ellipsoid, aseptate conidia (Verkley et al., 2014; Jayasiri et al., 2019). Thus, we report this new collection as a new host record of *P*. *archidendri* from dead twigs of *Magnolia* sp. in China.

**Figure 6:**
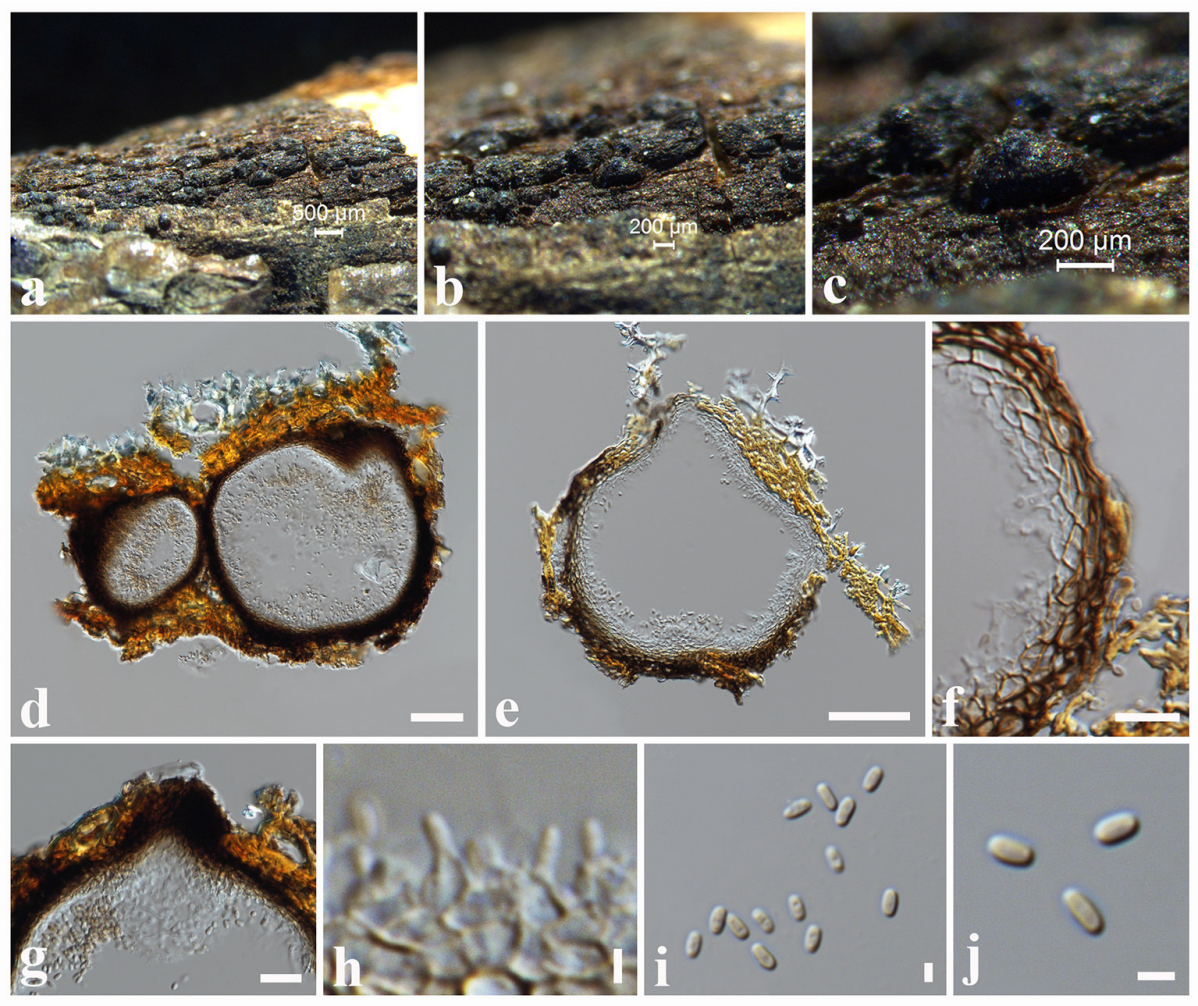
*Paraconiothyrium brasiliense* (MFLU 18-2649, new host record): **(a,b)** Conidiomata on a dead twig of *Magnolia* sp. **(c)** Close-up of conidiomata. **(d,e)** Sections through conidiomata. **(f)** Conidiomatal wall. **(g)** Sections through conidiomata showing neck region. **(h)** Conidiogenous cells and developing conidia. **(i,j)** Conidia. Scale bars: **(a)** = 500 μm, **(b,c)** = 200 μm, **(d,e)** = 50 μm, **(f)** = 10 μm, **(g)** = 20 μm, **(h–j)** = 2 μm.

***Paraconiothyrium brasiliense*** Verkley, Stud. Mycol. 50: 329 (2004) (Figure 6).

Index Fungorum Number: IF500082.

Facesoffungi number: FoF 00053.

*Saprobic* on dead twigs of *Magnolia* sp. (*Magnoliaceae*). **Sexual morph**: Undetermined. **Asexual morph**: Coelomycetous. *Conidiomata* 170–230 μm high, 180–250 μm diam. ($\bar{x}$ = 200 × 210 μm, *n* = 10), pycnidial, superficial or semi-immersed, dark brown to black, solitary to aggregated, globose to sub-globose. *Conidiomatal wall* 15–20 μm wide, outer layer comprising cells of *textura angularis* with somewhat thickened, brown walls, inner layer comprising flattened, hyaline, thin-walled cells of *textura angularis*. *Conidiophores* reduced to conidiogenous cells. *Conidiogenous cells* 4–5 × 1–2 μm ($\bar{x}$ = 4.6 × 1.5 μm, *n* = 20), discrete or integrated in short, formed from the inner cells all over the conidiomatal wall, simple, broadly ampulliform to globose, holoblastic, phialidic, hyaline to pale yellow. *Conidia* 2–4 × 1–2 ($\bar{x}$ = 3 × 1.5 μm, *n* = 40) μm, variable in shape, ellipsoid to short-cylindrical or obovoid, hyaline at immature, light brown at maturity, aseptate, smooth, rounded at both ends.

Culture characteristics: *Colonies* growing on PDA, 26 mm diam. after 3 weeks at 25°C, colonies circular, dense, smooth surface, undulate edge; from above: pale brown; reverse: brown.

Known hosts: *Acer pentaphyllum, Alliaria petiolate, Cinnamomum camphora, Coffea arabica, Ginkgo biloba, Magnolia* sp., *Pinus tabulaeformis, Picea glauca* and *Prunus salicina* (Damm et al., 2008; Paul and Lee, 2014; Verkley et al., 2014; Nakashima et al., 2019; this study).

Known distribution: Brazil, Canada, China, Japan, Korea, South Africa and the USA (Damm et al., 2008; Paul and Lee, 2014; Verkley et al., 2014; Nakashima et al., 2019; this study).

Material examined: China, Yunnan Province, Xishuangbanna, on dead twigs of *Magnolia* sp. (*Magnoliaceae*), 27 March 2017, N.I. de Silva, NI270 (MFLU 18-2649), living culture, MFLUCC 19-0040.

Notes: Phylogenetic analyses of a concatenated LSU, ITS, and *tub2* sequence data show that our strain (MFLUCC 19-0040) forms a well-supported clade, grouping with several strains of *Paraconiothyrium brasiliense* (80% ML, 0.99 BYPP, Figure 4). In particular, our strain provides a close phylogenetic relationship (87% ML, 0.95 BYPP) with the type of *P. brasiliense* (CBS 122851) and another strain of *P. brasiliense* (CBS 1223200). *Paraconiothyrium brasiliense* was described from fruits of *Coffea arabica* in Brazil (Verkley et al., 2014). This species shows widespread distribution and found on a wide range of host plants (Damm et al., 2008). *Paraconiothyrium brasiliense* was isolated as endophytes from *Ginkgo biloba* (Damm et al., 2008), *Acer pentaphyllum* in Korea (Paul and Lee, 2014), and healthy twig of *Cinnamomum camphora* in Japan (Nakashima et al., 2019). *P. brasiliense* was found associated with necrotic symptoms on *Prunus salicina* in South Africa (Damm et al., 2008). In this study, we identify our saprobic strain (MFLUCC 19-0040) as *P*. *brasiliense* and report its occurrence on *Magnolia* sp. in China for the first time.

**Figure 7:**
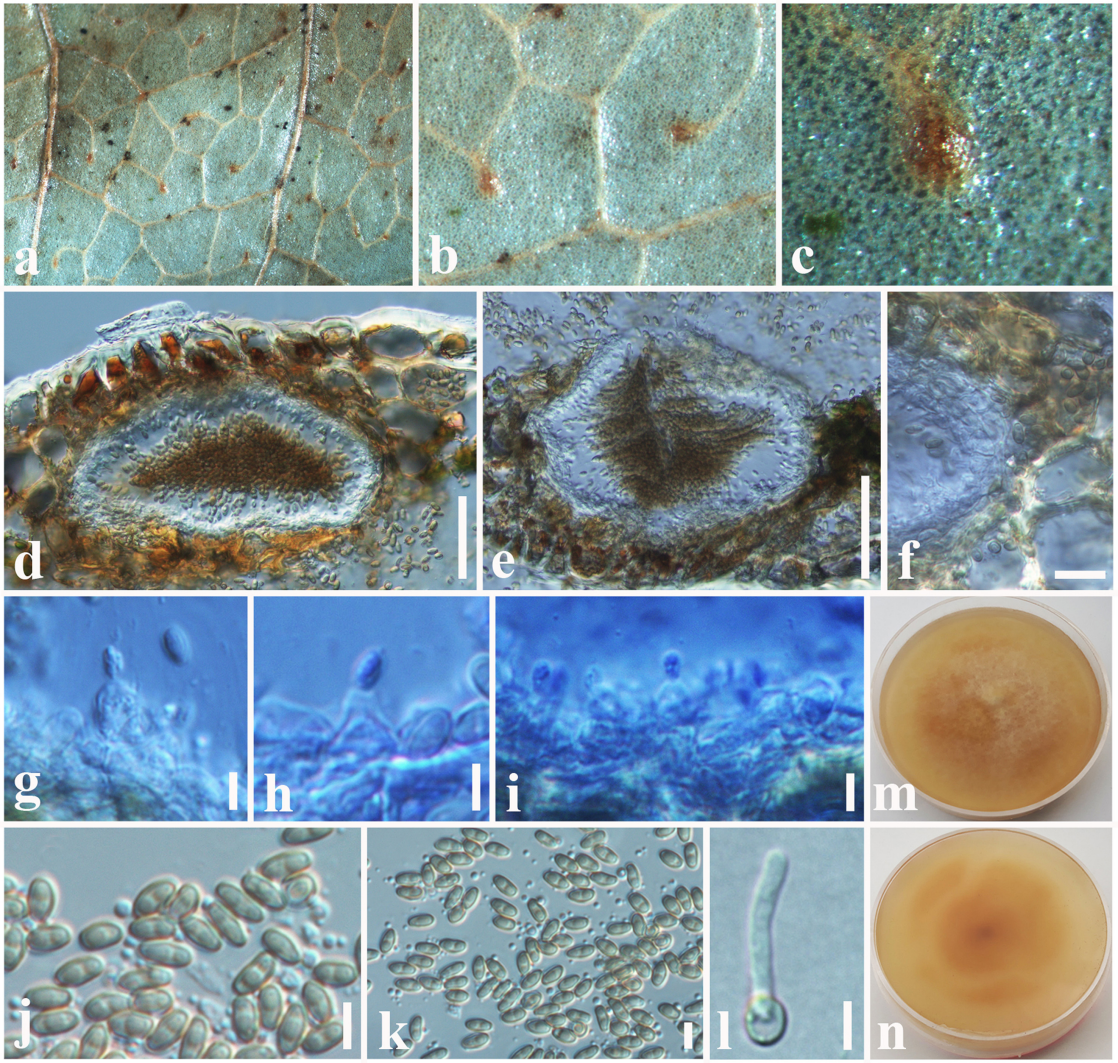
*Paraconiothyrium fuckelii* (MFLU 18-2605, new host record): **(a,b)** Conidiomata on a dead leaf of *Dimocarpus longan*. **(c)** Close-up of conidioma. **(d,e)** Sections through conidiomata. **(f)** Conidiomatal wall. **(g–i)** Conidiogenous cells and developing conidia. **(j,k)** Conidia. **(l)** Germinating conidium. **(m)** Upper side of the culture. **(n)** Lower side of the culture. Scale bars: **(d,e)** = 100 μm, **(f)** = 10 μm, **(g–l)** = 3 μm.

***Paraconiothyrium fuckelii*** (Sacc.) Verkley and Gruyter, Stud. Mycol. 75: 25 (2012) (Figure 7).

Index Fungorum Number: IF564787.

Facesoffungi number: FoF 00055.

≡ *Coniothyrium fuckelii* Sacc., Fungi venet. nov. vel. Crit., Sér. 5: 200 (1878).

= *Paraconiothyrium rosae* Senan., Wanas., Camporesi and K.D. Hyde, Fungal Divers. 31 (2018).

*Saprobic* on dead leaves of *Dimocarpus longan* Lour. (*Sapindaceae*). **Sexual morph**: Undetermined. **Asexual morph**: Coelomycetous. *Conidiomata* 150–300 μm high, 250–500 μm diam. ($\bar{x}$ = 240 × 320 μm, *n* = 10), pycnidial, immersed, light brown to dark brown, solitary, globose to sub-globose. *Conidiomatal wall* 15–20 μm wide, outer layer composed of somewhat thickened, brown-walled cells of *textura angularis*, inner layer composed hyaline, flattened, thin-walled cells of *textura angularis*. *Conidiophores* reduced to conidiogenous cells. *Conidiogenous cells* 4–8 × 3–5 μm ($\bar{x}$ = 5.2 × 4 μm, *n* = 20), discrete or integrated in short, simple, broadly ampulliform to globose, holoblastic, often annellidic, hyaline. *Conidia* 3–4 × 1–2.6 ($\bar{x}$ = 3.5 × 1.5 μm, *n* = 40) μm, variable in shape, sub-globose to ellipsoid or obovoid, rarely cylindrical, hyaline at immature, light brown at maturity, aseptate, smooth-walled.

Culture characteristics: *Colonies* growing on PDA, 15–20 mm diam. after 3 weeks at 25°C, colonies circular, slightly dense, smooth surface, crenate edge, velvety; from above: yellowish brown at the margin, brown in the center; reverse: yellowish brown at the margin, light brown in the center, mycelium white to whitish cream.

Known hosts: *Dimocarpus longan, Picea abies, Prunus* sp., *Punica granatum, Rosa hybrid*, and *Rubus* sp. (De Gruyter et al., 2006; Verkley et al., 2014; Gafforov, 2017; Jamali, 2020; this study).

Known distribution: China, Denmark, Germany, Iran, Netherlands, Thailand and Uzbekistan (De Gruyter et al., 2006; Ariyawansa et al., 2014a,b; Verkley et al., 2014; Gafforov, 2017; this study).

Material examined: China, Taiwan region, Chiayi, Ali Shan Mountain, on dead leaves of *Dimocarpus longan* (*Sapindaceae*), 05 September 2018, D.S. Tennakoon, TAP020 (MFLU 18-2605); living culture, MFLUCC 19-0067.

Notes: *Paraconiothyrium fuckelii* was first introduced by Saccardo (1876) from cane lesions on raspberry, similar to *Coniothyrium fuckelii*. Subsequently, *C. fuckelii* was synonymized under *Paraconiothyrium* by De Gruyter et al. (2006), based on morphology combined with phylogeny data. The morphological characteristics of our collection (MFLU 18-2605) resemble previously introduced authentic *P. fuckelii* isolates in having pycnidial, immersed, light brown to dark brown, globose to sub-globose conidiomata, ampulliform to globose, holoblastic conidiogenous cells and sub-globose to ellipsoid or obovoid, hyaline to light brown, aseptate conidia (Ariyawansa et al., 2014b; Verkley et al., 2014). Multi-gene phylogeny also directs that our collection (MFLU 18-2605) groups with other *P. fuckelii* isolates in a highly supported clade (97% ML, 0.99 BYPP, Figure 4). In particular, our isolate shows a close phylogeny relationship (99% ML, 1.00 BYPP) with the isolate MFLUCC 13-0073, which was introduced by Ariyawansa et al. (2014b) from Thailand (Figure 4). Hence, we consider MFLU 18-2605 as a new host record of *P. fuckelii* from *Dimocarpus longan* (*Sapindaceae*). *Paraconiothyrium fuckelii* seems to have a cosmopolitan distribution since it has been reported from different plant families, such as *Fabaceae, Rosaceae, Pinaceae*, and *Sapindaceae* worldwide (Kuter, 1986; De Gruyter et al., 2006; Mułenko et al., 2008; Kowalski and Andruch, 2012; Ariyawansa et al., 2014b; Verkley et al., 2014). Many researchers have identified the asexual morph of this species and a sexual morph introduced by Ariyawansa et al. (2014b), which has immersed to erumpent, globose or sub-globose, coriaceous, ostiolate ascomata; clavate asci; and pale brown, 3-septate, narrowly ovoid to clavate ascospores.

In addition, *P. rosae* was introduced by Wanasinghe et al. (2018) from dead aerial spines of *Rosa canina* (*Rosaceae*) without statistical support. In the present phylogenetic analyses, *P. rosae* groups well with *P. fuckelii* isolates in a highly supported clade (97% ML, 0.99 BYPP, Figure 4). The morphological characteristics of *P. rosae* also agree well with *P. fuckelii* in having pycnidial, immersed or semi-immersed, globose to sub-globose conidiomata and sub-globose to ellipsoid or obovoid, hyaline to light brown, aseptate conidia (Ariyawansa et al., 2014b; Verkley et al., 2014; Wanasinghe et al., 2018). Therefore, we treat *P. rosae* as a synonym of *P*. *fuckelii* in this study.

**Figure 8:**
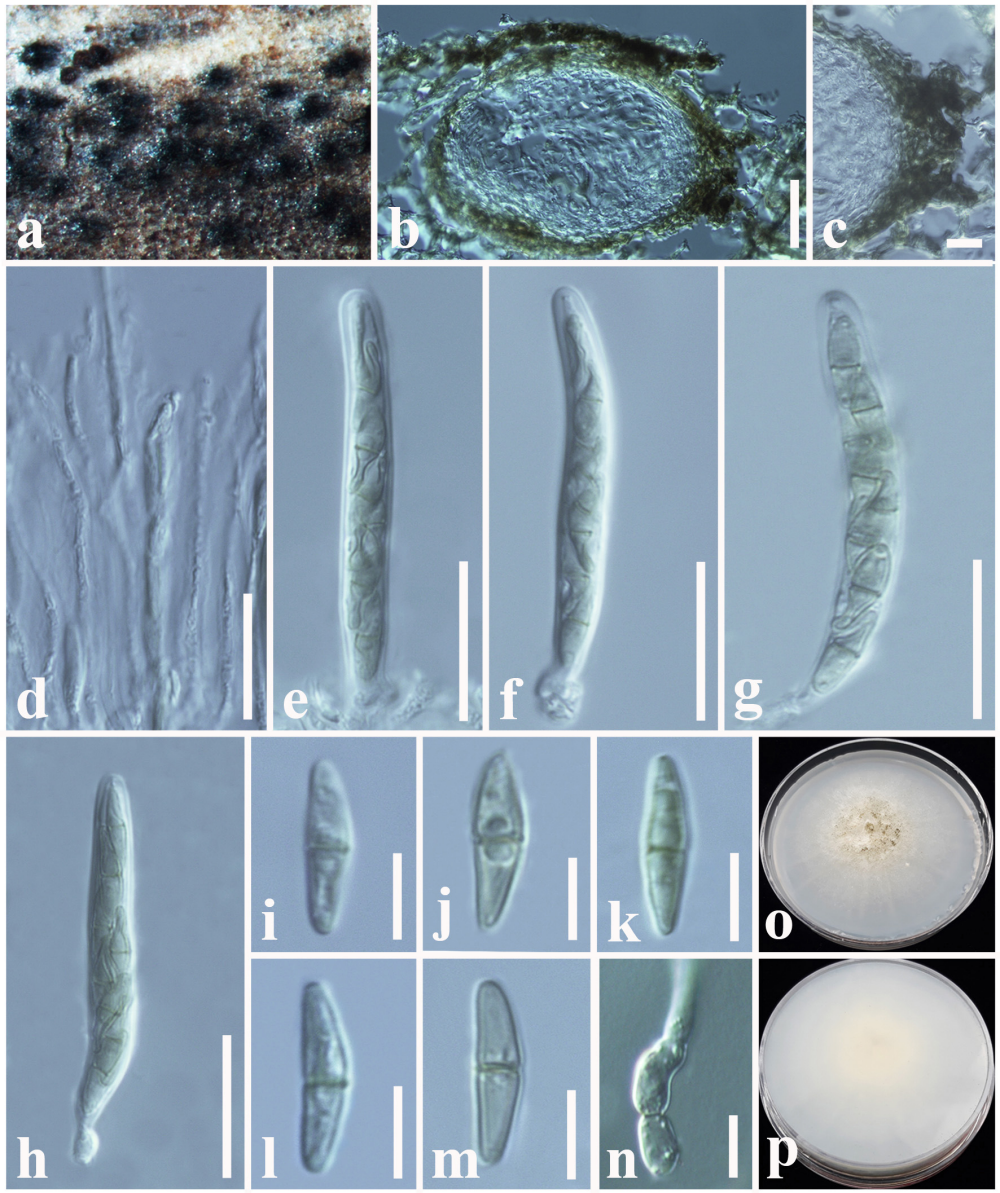
*Paraconiothyrium zingiberacearum* (NCYU 19-0320, holotype). **(a)** Ascomata on a dead stem of *Hedychium coronarium*. **(b)** Vertical section through an ascoma. **(c)** Peridium. **(d)** Pseudoparaphyses. **(e–h)** Asci. **(i–m)** Ascospores. **(n)** Germinating ascospore. **(o)** Upper side of the culture. **(p)** Lower side of the culture. Scale bars: **(b)** = 50 μm, **(c)** = 10 μm, **(d–h)** = 20 μm, **(i–n)** = 6 μm.

***Paraconiothyrium zingiberacearum*** Tennakoon and S. Lumyong, sp. nov. (Figure 8).

Index Fungorum Number: IF559600.

Facesoffungi number: FoF 10802.

Etymology: The species name reflects the host family *Zingiberaceae*, from which the holotype was collected.

Holotype: NCYU 19-0320.

*Saprobic* on dead stem of *Hedychium coronarium* J. Koenig (*Zingiberaceae*). **Sexual morph**: *Ascomata* 100–170 μm high × 120–220 μm diam. ($\bar{x}$ = 150 × 180 μm, *n* = 10), solitary or clustered, coriaceous, immersed to semi-immersed, erumpent, globose to sub-globose, unilocular, brown to dark brown. *Peridium* 20–30 μm wide, outer layer containing light brown to dark brown, thick-walled, tightly packed cells of *textura angularis*, inner layer containing flattened, hyaline, thin-walled, loosely packed cells of *textura angularis. Hamathecium* comprising 1.5–3 μm wide, cylindrical to filiform, septate, cellular pseudoparaphyses. *Asci* 50–60 × 5.5–7 μm ($\bar{x}$ = 53 × 6.5 μm, *n* = 20), bitunicate, 8-spored, fissitunicate, clavate to cylindrical, apex rounded, short pedicel, with a shallow ocular chamber. *Ascospores* 11–14 × 2.5–4 μm ($\bar{x}$ = 12.5 × 3 μm, *n* = 30), overlapping, 1–2-seriate, light brown, broadly fusiform with acute ends, 1-septate, slightly curved, rough-walled, guttulate, without a mucilaginous sheath. **Asexual morph**: Undetermined.

Culture characteristics: *Colonies* growing on PDA, 10–12 mm diam. after 3 weeks at 25°C, colonies circular, slightly dense, surface smooth, crenate edge, cottony; from above: white to cream at the margin, cream to gray in the center; reverse: white at the margin, light brown to yellowish in the center.

Material examined: China, Taiwan region, Chiayi, on a dead stem of *Hedychium coronarium* (*Zingiberaceae*), 05 August 2017, D.S. Tennakoon, NSP002a (NCYU 19-0320, holotype); ex-type living culture, NCYUCC 19-0230. *ibid*. 08 August 2017, NSP002b (MFLU 18-0091, Paratype); ex-paratype living culture, MFLUCC 18-0559.

Notes: Phylogenetic analyses indicate that our collection (MFLU 18-0091 and NCYU 19-0320) is grouped with *Paraconiothyrium estuarinum* with high statistical support (89% ML, 0.96 BYPP, Figure 4). The asexual morph of *P. estuarinum* was introduced by Verkley et al. (2004) from an estuarine sediment polluted with industrial discharges in Brazil. However, we were unable to compare our collection with the asexual morph of *P. estuarinum* (CBS 109850) because it failed to sporulate even after 2 months of the incubation period (Verkley et al., 2004). Therefore, we compared the base pair differences between our collection and *P. estuarinum*. There are 10 base pair differences (2.21%) across 451 nucleotides across the *tub2* gene region and 11 base pair differences (2.17%) across 506 nucleotides across the ITS (+5.8S) gene region. Phylogeny also indicates that the clade containing our collection and *P. estuarinum* provides sister lineage to *P*. *cyclothyrioides* (Figure 4). The sexual morph of *P. cyclothyrioides* differs from our collection in having olive-brown, 3-septate, cylindrical to ellipsoidal ascospores with obtusely rounded ends, whereas our collection has light brown, 1-septeate, broadly fusiform ascospores with acute ends (Hyde et al., 2020a). Hence, based on significant differences, we present our collection as a novel species, *P. zingiberacearum* from dead stems of *Hedychium coronarium* (*Zingiberaceae*).

### Phylogenetic and taxonomic results of *Paraphaeosphaeria*

#### *Paraphaeosphaeria* O. E. Erikss

Notes: *Paraphaeosphaeria* was established by Eriksson (1967) to include four species, which have oblong-cylindric ascospores. The sexual morphs of *Paraphaeosphaeria* have immersed or semi-immersed ascomata, short pedicellate asci, and broadly elliptical, yellowish brown, multi-septate ascospores (Ariyawansa et al., 2014b; Hongsanan et al., 2020). Asexual morphs have pycnidial conidiomata and aseptate or 1-septate conidia (Wanasinghe et al., 2018; Hongsanan et al., 2020). Ariyawansa et al. (2014b) verified the phylogenetic placement of this genus in *Didymosphaeriaceae*. Currently, 30 species epithets are listed under *Paraphaeosphaeria* in Index Fungorum (2022). In this study, we introduce a new species (*P. brachiariae*) from dead leaves of *Brachiaria mutica*.

**Figure 9:**
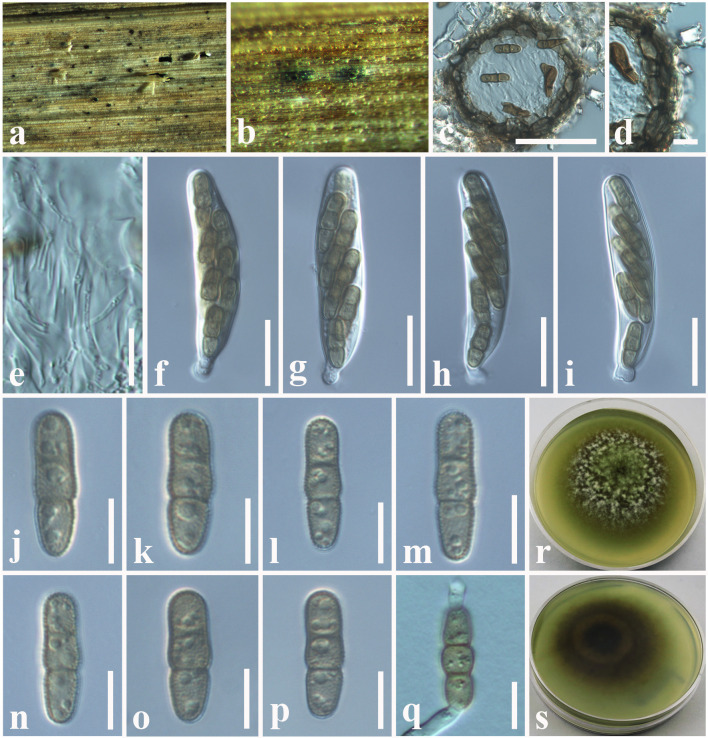
*Paraphaeosphaeria brachiariae* (NCYU 19-0058, holotype). **(a,b)** Ascomata on a dead leaf of *Brachiaria mutica*. **(c)** Vertical section through an ascoma. **(d)** Peridium. **(e)** Pseudoparaphyses. **(f–i)** Asci. **(j–p)** Ascospores. **(q)** Germinating ascospore. **(r)** Upper side of the culture. **(s)** Lower side of the culture. Scale bars: **(c)** = 50 μm, **(d)** = 10 μm, **(e–i)** = 20 μm, **(j–q)** = 7 μm.

***Paraphaeosphaeria brachiariae*** Tennakoon and S. Lumyong, sp. nov. (Figure 9).

Index Fungorum Number: IF559601

Facesoffungi number: FoF 10803

Etymology: Name reflects the host genus, *Brachiaria*.

Holotype: NCYU 19-0058

*Saprobic* on dead leaves of *Brachiaria mutica* (Forssk.) Stapf. (*Poaceae*). **Sexual morph**: *Ascomata* 60–120 μm high × 50–100 μm diam. ($\bar{x}$ = 75 × 80 μm, *n* = 10), solitary, scattered, coriaceous, immersed to semi-immersed, partly erumpent, unilocular, globose to sub-globose, brown to dark brown, ostiolate, with minute papilla, filled with hyaline cells. *Peridium* 10–20 μm wide, comprising light brown to dark brown, thick-walled, loosely packed cells of *textura angularis*. *Hamathecium* comprising 1.5–2.5 μm wide, cylindrical to filiform, branched, septate, pseudoparaphyses. *Asci* 58–65 × 11–13 μm ($\bar{x}$ = 62 × 12.5 μm, *n* = 20), bitunicate, 8-spored, fissitunicate, clavate to cylindrical, apex rounded, short pedicellate, with a shallow ocular chamber. *Ascospores* 14–20 × 4–6 μm ($\bar{x}$ = 16 × 5 μm, *n* = 30), overlapping, 1–2-seriate, light brown, fusiform with rounded ends, straight or slightly curved, with 1–2-septate, guttulate, rough-walled, without a mucilaginous sheath. **Asexual morph**: Undetermined.

Culture characteristics: *Colonies* growing on PDA, 12–15 mm diam. after 3 weeks at 25°C, colonies circular, smooth surface, crenate edge, cottony; from above: light brown to greenish at the margin, gray in the center; reverse: light brown to yellowish at the margin, brown in the center, mycelium greenish to whitish gray.

Material examined: China, Taiwan region, Chiayi, on dead leaves of *Brachiaria mutica* (*Poaceae*), 10 July 2019, D.S. Tennakoon, PC058A (NCYU 19-0058, holotype); ex-type living culture, NCYUCC 19-0342. *ibid*. 18 July 2019, PC058A (MFLU 19-2799, Paratype); paratype living culture, NCYUCC 19-0343.

Notes: The morphological characteristics of our collection (MFLU 19-2799 and NCYU 19-0058) agree well within the species concept of *Paraphaeosphaeria* in having immersed or semi-immersed ascomata, short pedicellate asci, and broadly elliptical, multi-septate, yellowish brown ascospores (Ariyawansa et al., 2014b; Hongsanan et al., 2020). Phylogeny (LSU, SSU, ITS, and *tub2*) shows that our collection (MFLU 19-2799 and NCYU 19-0058) grouped with *P. angularis* with high statistical support (99% ML, 1.00 BYPP). The asexual morph of *P. angularis* was introduced by Verkley et al. (2014) from *Saccharum officinarum* in Brazil. A comparison of 539 nucleotides across the ITS (+5.8S) gene region of *P. angularis* (CBS 167.70) and our collection (MFLU 19-2799) shows 11 base pair differences (2.04%). We compared 452 nucleotides across the *tub2* gene as well, and there were 12 base pair differences (2.65%). Thus, we present our collection as a new species, *P. brachiariae* from *Brachiaria mutica*.

### Phylogenetic and taxonomic results of *Spegazzinia*

#### *Spegazzinia* Sacc

Notes: Saccardo (1880) established *Spegazzinia* to include *S. ornata* as the type species. This genus was previously placed in *Apiosporaceae* (*Sordariomycete*s) by Hyde et al. (1998), based on its morphological characteristics. Subsequently, its phylogenetic placement was revealed by Tanaka et al. (2015) and accommodated in *Didymosphaeriaceae* (*Dothideomycetes*). *Spegazzinia* has a diverse distribution worldwide as saprobes on dead materials of vascular plants (Leão-Ferreira and Gusmão, 2010; Manoharachary and Kunwar, 2010; Thambugala et al., 2017; Samarakoon et al., 2020; Tennakoon et al., 2021), endophytes from lichens, and plant leaves (Crous et al., 2019), and even they have been recorded from soil (Ellis, 1971). The conidial morphology and basauxic conidiogenesis of *Spegazzinia* differentiate it apart from other dematiaceous hyphomycetes. The majority has distinct morphological characteristics in the shapes of α and β conidia (Hongsanan et al., 2020). A total of 14 *Spegazzinia* species are currently listed in Index Fungorum (2022). In this study, we introduce two new host records of *Spegazzinia deightonii* and *S*. *tessarthra* from *Hedychium coronarium* and *Acacia auriculiformis*, respectively.

**Figure 10:**
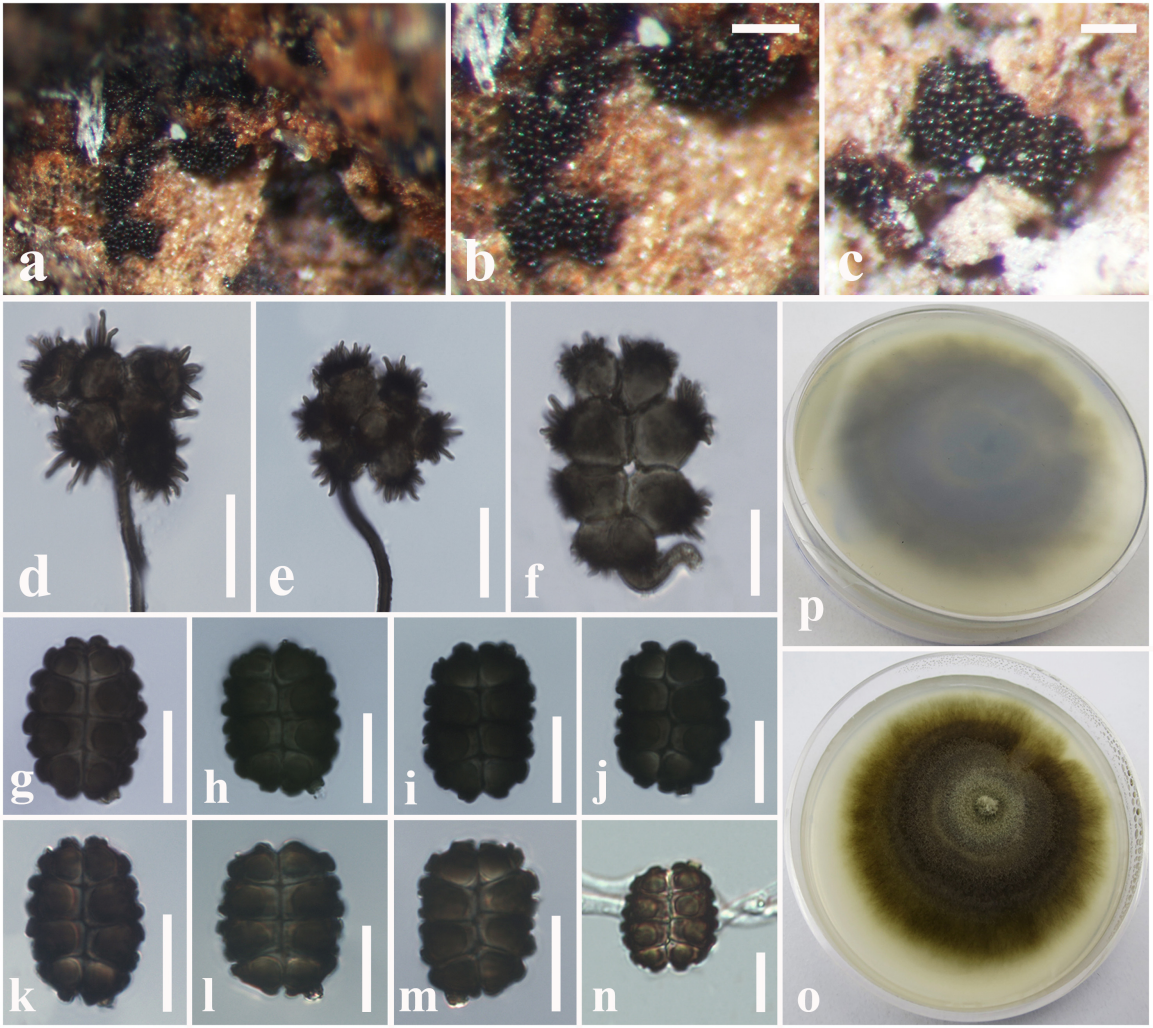
*Spegazzinia deightonii* (MFLU 18-2564, new host record). **(a–c)** Fungal colonies on a dead stem of *Hedychium coronarium*. **(d,e)** α Conidia with conidiophores. **(f)** α Conidium with conidiogenous cell. **(g–m)** β Conidia. **(n)** A germinating β conidium. **(o)** Upper side of the culture. **(p)** Lower side of the culture. Scale bars: **(b,c)** = 20 mm, **(d–n)** = 10 μm.

***Spegazzinia deightonii*** (S. Hughes) Subram., J. Indian Bot. Soc. 35: 78 (1956) (Figure 10).

Index Fungorum number: IF306062.

Facesoffungi number: FoF 07238.

*Saprobic* on dead stem of *Hedychium coronarium* J. Koenig (*Zingiberaceae*). **Sexual morph**: Undetermined. **Asexual morph**: Hyphomycetous. *Sporodochia* 1–2 mm diam., dense, dark, velvety, powdery. *Conidiophores* long and micronematous. *Conidiophores* of α conidia up to 50–100 × 1–2 μm ($\bar{x}$ = 78 × 1.5 μm, *n* = 20) long, light brown to dark brown, verruculose, narrow, erect or flexuous, unbranched. *Conidiogenous cells* basauxic, verrucose, pale brown. *Conidia* holoblastic, two types, α *conidia* 17–27 × 18–26 μm ($\bar{x}$ = 24 × 22 μm, *n* = 25), stellate, solitary, globose to variously shaped, with spines 4–5 μm long, 4–8-celled, frequently 4–6-celled, deeply constricted at the septa. β *conidia* 15–20 × 10–15 μm ($\bar{x}$ = 18 × 13 μm, *n* = 25), hyaline at immature, light brown to dark brown at mature, 8-celled, disc-shaped, both sides slightly flat with short and blunt spines.

Culture characteristics: *Colonies* growing on PDA, 15–20 mm diam. after 3 weeks at 25°C, colonies circular, slightly dense, surface smooth, velvety; from above: gray to light brown at the margin, dark gray to brown in the center; reverse: yellowish brown at the margin, dark brown to black in the center, mycelium greenish to light brown.

Known hosts: *Areca catechu, Cocos nucifera, Hedychium coronarium, Musa* sp. and *Panicum maximum* (Matsushima, 1980; Lu et al., 2000; Tianyu, 2009; Samarakoon et al., 2020; Farr and Rossman, 2022; this study).

Known distribution: China, and Thailand (Matsushima, 1980; Lu et al., 2000; Tianyu, 2009; Samarakoon et al., 2020; Farr and Rossman, 2022; this study).

Material examined: Thailand, Chiang Rai, a dead stem of *Hedychium coronarium* (Z*ingiberaceae*), 11 July 2018, D.S. Tennakoon, DP017 (MFLU 18-2564); living culture, MFLUCC 18-1625.

**Figure 11:**
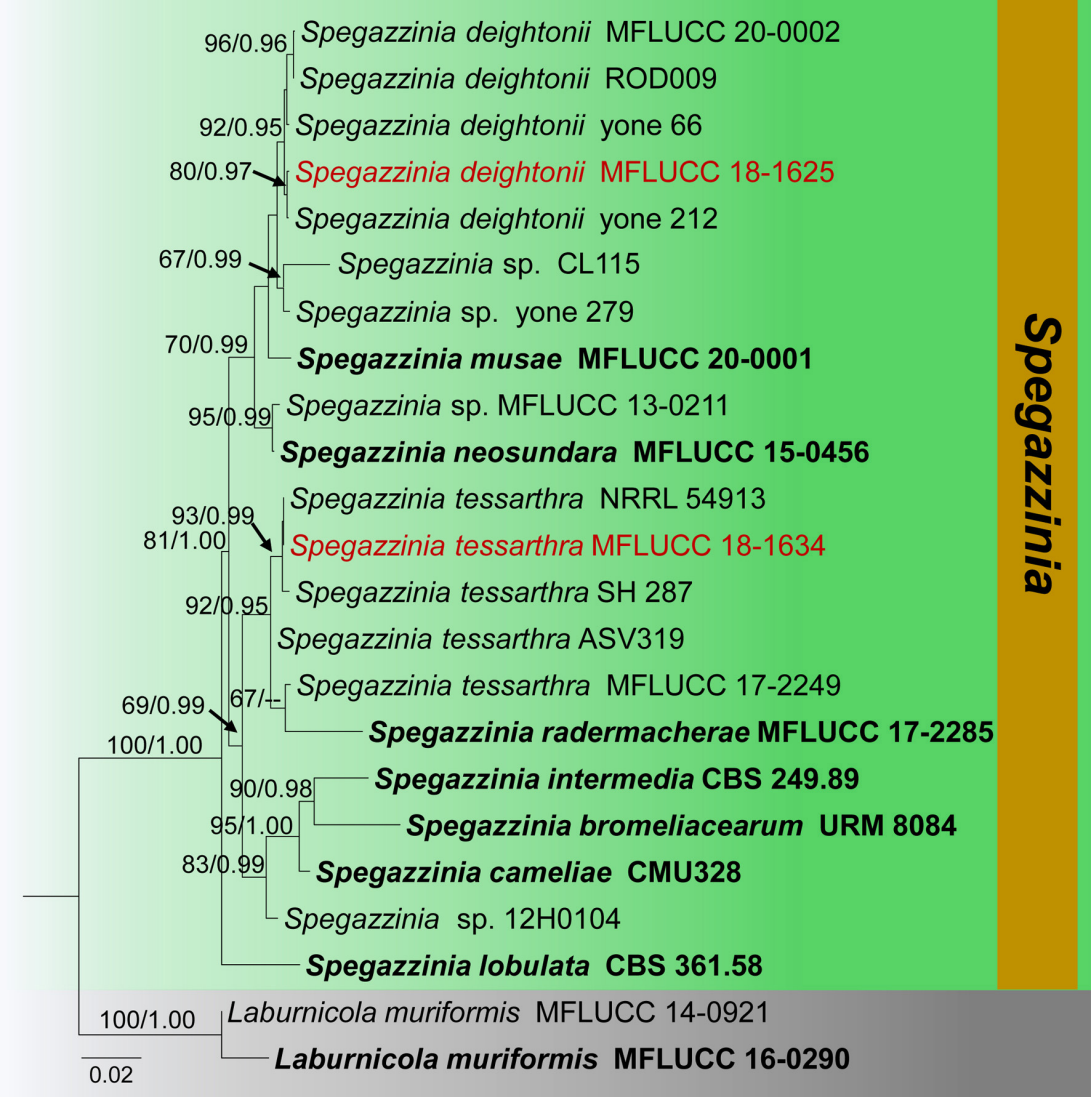
Phylogram generated from maximum likelihood analysis is based on combined ITS, LSU, SSU, and *tef1-*α sequence data (Final likelihood value of −8278.609544). The tree is rooted with *Laburnicola muriformis* (MFLUCC 14-0921, MFLUCC 16-0290). The ex-type strains are indicated in bold, and the new strains are indicated in red. Bootstrap support values ≥65% of maximum likelihood (ML) and Bayesian posterior probabilities (PP) values ≥0.90 are given above the nodes.

Notes: Due to the high similarities of morphological characteristics and phylogenetic affinities, we introduce our collection (MFLU18-2564) as a new host record of *S. deightonii* from *Hedychium coronarium* (*Zingiberaceae*) in Thailand. Phylogenetic analyses of this study indicate that our collection is nested with other *S. deightonii* isolates in a well-supported clade (92% ML, 0.95 BYPP) (Figure 11). As well as, our collection (MFLU 18-2564) shows a close phylogenetic relationship with the isolate yone 212, which was introduced by Tanaka et al. (2015) from Japan. Both isolates share similar morphological characteristics, such as 8-celled, disked-shaped, dark brown, spiny conidia (Tanaka et al. 2015). *Spegazzinia deightonii* has previously been recorded from many host species (e.g., *Areca catechu, Cocos nucifera, Musa* sp., and *Panicum maximum*), and this is the first host record from *Hedychium coronarium* (*Zingiberaceae*) in Thailand.

**Figure 12:**
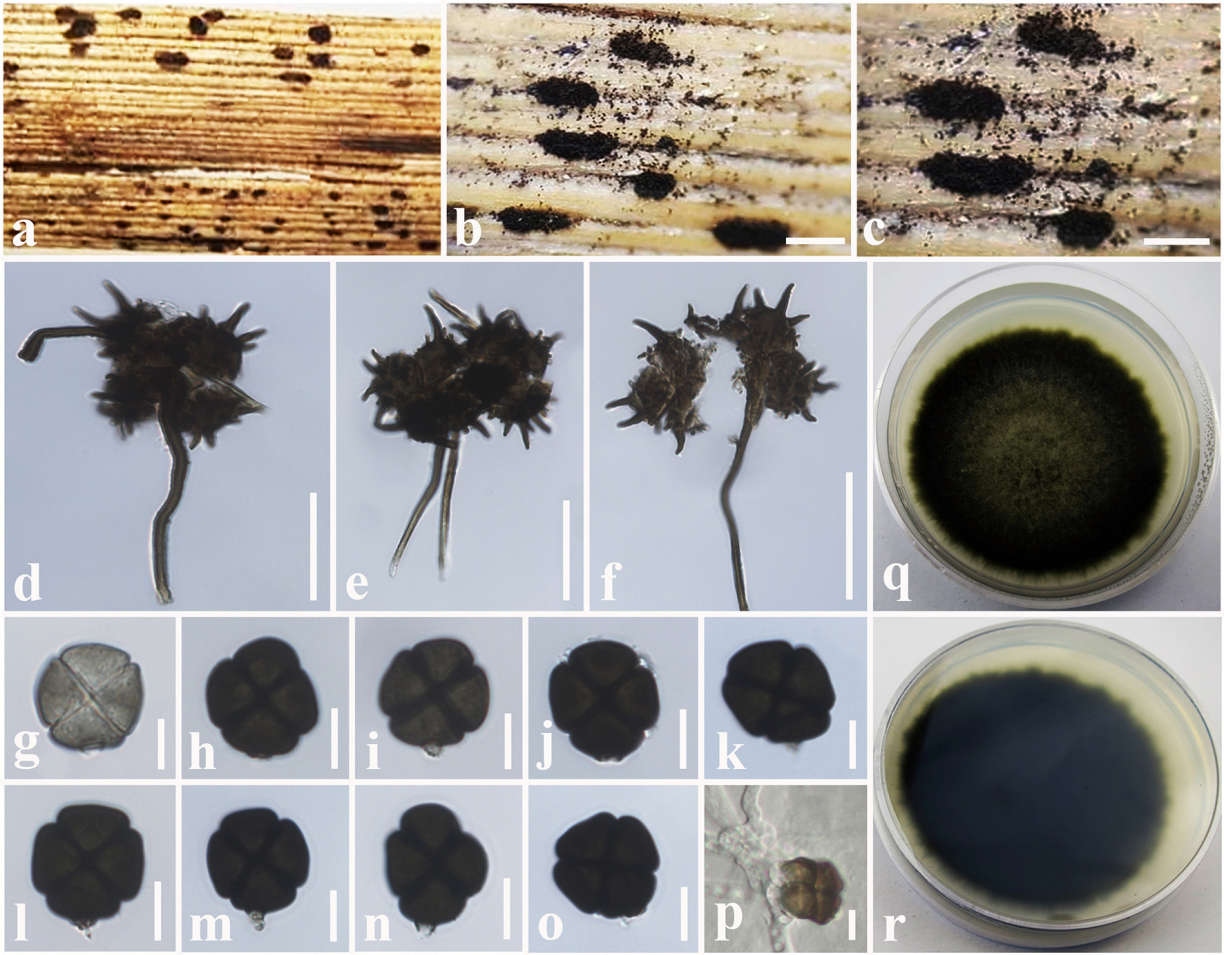
*Spegazzinia tessarthra* (MFLU 18-2557, new host record). **(a–c)** Fungal colonies on a dead stem of *Acacia auriculiformis*. **(d–f)** α Conidia with conidiophores. **(g**–**o)** β Conidia. **(p)** A germinating β conidium. **(q)** Upper side of the culture. **(r)** Lower side of the culture. Scale bars: **(b,c)** = 2 mm, **(d–f)** = 30 μm, **(g–p)** = 6 μm.

***Spegazzinia tessarthra*** (Berk. and M.A. Curtis) Sacc., Syll. Fung. (Abellini) 4: 758 (1886) (Figure 12).

Index Fungorum Number: IF219777.

Facesoffungi number: FoF 08241.

= *Sporidesmium tessarthrum* Berk. and M.A. Curtis,. Soc., Bot. 10(no. 46): 355 (1868).

*Saprobic* on dead stem of *Acacia auriculiformis* A. Cunn. (*Fabaceae*). **Sexual morph**: Undetermined. **Asexual morph**: Hyphomycetous. *Sporodochia* 1–3 mm diam., dense, dark, powdery, velvety. *Conidiophores* of α conidia up to 30–60 × 1.5–2.5 μm ($\bar{x}$ = 48 × 1.8 μm, *n* = 15), erect or flexuous, pale brown to dark brown, rough-walled, narrow and long, generally unbranched. *Conidiogenous cells* basauxic, verrucose, pale brown. *Conidia* solitary, two types, α conidia stellate, 15–20 × 14–18 μm ($\bar{x}$ = 17 × 16 μm, *n* = 20), globose to sub-globose, 4–6-celled, deeply constricted at the septa, conspicuously spinulate, 4–6 spines, each spine 2–8 μm long. β conidia 10–15 × 8–12 μm ($\bar{x}$ = 12 × 11 μm, *n* = 20), disc-shaped, initially hyaline, becoming light brown to dark brown at maturity, 4-celled, rough-walled, crossed septate, smooth-walled, somewhat obovoid, deeply constricted at the septa, dark brown in constricted areas, flat from side view.

Culture characteristics: *Colonies* growing on PDA, 10–12 mm diam. after 3 weeks at 25°C, colonies circular, dense, smooth surface, entire edge, cottony; from above: light brown to yellowish at the margin, dark brown to black in the center; reverse: yellowish brown at the margin, dark brown to black in the center, mycelium dark brown to dark gray.

Known hosts: *Acacia auriculiformis, Ochroma pyramidale* and *Zea mays* (Berkeley and Curtis, 1869; Tanaka et al., 2015; Farr and Rossman, 2022; this study).

Known distribution: Cuba, India, Japan, and Thailand (Berkeley and Curtis, 1869; Tanaka et al., 2015; Farr and Rossman, 2022; this study).

Material examined: Thailand, Ching Rai, a dead stem of *Acacia auriculiformis* (*Fabaceae*), 15 July 2018, D.S. Tennakoon, DP003 (MFLU 18-2557); living culture, MFLUCC 18-1624.

Notes: New collection (MFLU 18-2557) resembles *Spegazzinia tessarthra* (SH 287), which was introduced by Tanaka et al. (2015) from balsa wood in Japan. Both isolates have two types of conidia (α conidia: 4–6-celled, globose to sub-globose, 4–6 spines; β conidia: disc-shaped, 4-celled, light brown to dark brown) (Tanaka et al., 2015). Multi-gene (LSU, SSU, ITS, and *tef1-*α) phylogenetic analyses also indicate that our collection (MFLU 18-2557) groups with *S. tessarthra* isolates (NRRL 54913, SH 287) in a well-supported clade (93% ML, 0.99 BYPP, Figure 11). Hence, we consider MFLU 18-2557 as a new host record of *S. tessarthra* from *Acacia auriculiformis* (*Fabaceae*) in Thailand. This species has previously been recorded from *Acacia auriculiformis, Ochroma pyramidale*, and *Zea mays* (Berkeley and Curtis, 1869; Tanaka et al., 2015; Farr and Rossman, 2022).

## Discussion

*Didymosphaeriaceae* species has a great variation in morphologies, phylogenies, ecologies, and nutritional modes (Hyde et al., 2013; Ariyawansa et al., 2014b; Liu et al., 2015; Gonçalves et al., 2019; Htet et al., 2021; Suwannarach et al., 2021). It has a diverse range of nutritional modes, such as endophytes, pathogens, and saprobes in plant substrates (Zhang et al., 2012; Liu et al., 2015; Gonçalves et al., 2019; Suwannarach et al., 2021). Researchers have referred this family into several higher taxa due to the uncertainty of the taxonomic placement (von Arx and Müller, 1975; Lumbsch and Huhndorf, 2007; Crous et al., 2012; Zhang et al., 2012). For instance, this family was a synonym of *Pleosporaceae* (von Arx and Müller, 1975), and Barr (1990) referred *Didymosphaeriaceae* in *Melanommatales*. Aptroot (1995) assigned this as a separate family within *Pleosporales*, and Lumbsch and Huhndorf (2007) treated *Didymosphaeriaceae* members to the *Montagnulaceae* in their outline of *Ascomycota*. However, the confusion surrounding the genera of *Didymosphaeriaceae* and *Montagnulaceae* was debated by Ariyawansa et al. (2014a), and they pointed out *Didymosphaeriaceae* as a distinct family in *Pleosporales* upon morphology, but phylogenetically, it fits well with *Montagnulaceae*. Thus, Ariyawansa et al. (2014b) synonymized *Montagnulaceae* under *Didymosphaeriaceae* and accepted 16 genera in this family. Over time, numerous genera have been introduced, and currently, 32 genera are accepted in *Didymosphaeriaceae* (Hongsanan et al., 2020).

Various approaches have been used to identify *Didymosphaeriaceae* species over the years. Earlier, morphology-based species recognition was the key method for identifying *Didymosphaeriaceae* species, and most studies have been used drawings with descriptions (von Arx and Müller, 1975; Sutton, 1980; Barr, 1990). However, morphology-based species identification suffered various issues, including phenotypic plasticity, which may lead to countless misinterpretations. However, by using molecular techniques for species delineation, identification, and taxonomic classifications, this fungal taxonomy undergone a revolution (Ariyawansa et al., 2014a; Das et al., 2014; Chethana et al., 2020). Therefore, most of recent studies have integrated morphology and phylogeny data for *Didymosphaeriaceae* species identification, taxonomic classification, and phylogenetic inferences (Ariyawansa et al., 2014a,b; Wanasinghe et al., 2016; Mapook et al., 2020; Htet et al., 2021; Suwannarach et al., 2021). Apart from morphology and phylogeny classifications, some researchers have been carried out to estimate the divergence time of this family using molecular clock analyses (Khodaei et al., 2019), and some have investigated the secondary metabolites or biological functions of *Didymosphaeriaceae* species (Wang et al., 2021).

**Figure 13:**
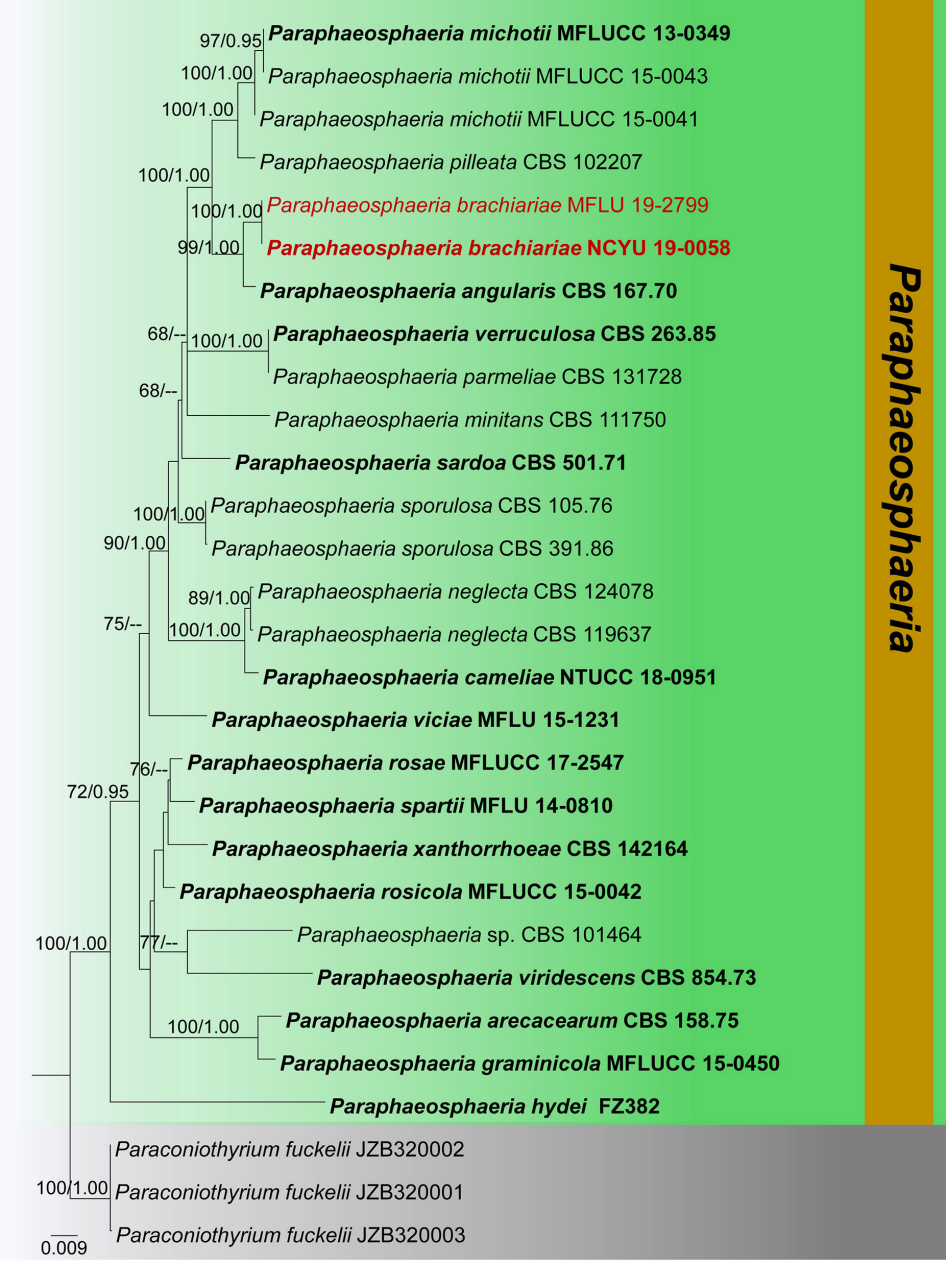
Phylogram generated from maximum likelihood analysis is based on combined ITS, LSU, SSU, and *tub2* sequence data (final likelihood value of −9171.108559). The tree is rooted with *Paraconiothyrium fuckelii* (JZB320001, JZB320002, and JZB320003). The ex-type strains are indicated in bold, and the new isolates are indicated in red. Bootstrap support values ≥65% of maximum likelihood (ML) and Bayesian posterior probabilities (PP) values ≥0.90 are given above the nodes.

The phylogeny recovered in this study agrees well with previous *Didymosphaeriaceae* studies within *Pleosporales* (Ariyawansa et al., 2014b; Wanasinghe et al., 2018; Phookamsak et al., 2019; Mapook et al., 2020; Phukhamsakda et al., 2020). The morphological characteristics of the novel species (*Montagnula acaciae, Paraconiothyrium zingiberacearum*, and *Paraphaeosphaeria brachiariae*) fit well with the respective genera, are phylogenetically distinct from other species, and group with high statistical evidence (Figures 2, 4, 11, 13). Also, the morphology of the new host records (*Montagnula jonesii, Paraconiothyrium archidendri, P. brasiliense, P. fuckelii, Spegazzinia deightonii*, and *S*. *tessarthra*) also agrees with their respective type species (Saccardo, 1876; Ariyawansa et al., 2014a,b; Tanaka et al., 2015; Tennakoon et al., 2016). Overall, this work offers fascinating taxonomic insights into *Didymosphaeriaceae* species. In particular, the findings provide evidence to show the vast range of fungal diversity in litter substrates (e.g., dead leaves and dead stems), even within a single family, *Didymosphaeriaceae*. But, to determine whether these fungal species are generalists or specialists, more ecological studies are required. Furthermore, it is worthy to note that several *Didymosphaeriaceae* genera still lack molecular data to determine the phylogenetic placement (e.g., *Barria* and *Lineostroma*). Therefore, it is necessary to collect, isolate, and retrieve sequence data for more *Didymosphaeriaceae* species in future studies.

## Conclusion

Studies on fungal species in plant litter are particularly pertinent in the current climate change scenario since climate plays a crucial role in decomposition and, ultimately, nutrient cycling, soil fertility, and the global carbon cycle. This study revealed plant litter inhabiting three new fungal species (*Montagnula acaciae, Paraconiothyrium zingiberacearum*, and *Paraphaeosphaeria brachiariae*) and six new host records (*Montagnula jonesii, Paraconiothyrium archidendri, P*. *brasiliense, P*. *fuckelii, Spegazzinia deightonii*, and *S*. *tessarthra*) from China and Thailand. In addition, *Paraconiothyrium rosae* was synonymized under *P*. *fuckelii* based on morphology and phylogeny data. We believe that this study sheds light on the plant litter-dwelling fungal communities, which are diverse and astonishing.

## Data availability statement

The datasets presented in this study can be found in online repositories. The names of the repository/repositories and accession number(s) can be found in the article/supplementary material.

## Author contributions

DT, KT, and NIS: conceptualization, methodology, formal analysis, and writing—original draft preparation. DT and NIS: software and data curation. KT and NS: validation. KT and SL: investigation. DT: resources. KT, NIS, NS, and SL: writing—review and editing. NS and SL: supervision and funding acquisition. SL: project administration. All authors have read and agreed to the published version of the manuscript.

## Funding

This research was supported by the Post-Doctoral Fellowship 2022 for Reinventing Chiang Mai University (R000030885). This project was also funded by the National Research Council of Thailand (NRCT) N42A650198.

## Conflict of interest

The authors declare that the research was conducted in the absence of any commercial or financial relationships that could be construed as a potential conflict of interest. The reviewer SCK is currently organizing a Research Topic with the author NS.

## Publisher's note

All claims expressed in this article are solely those of the authors and do not necessarily represent those of their affiliated organizations, or those of the publisher, the editors and the reviewers. Any product that may be evaluated in this article, or claim that may be made by its manufacturer, is not guaranteed or endorsed by the publisher.
